# A Minimum 3‐Year Follow‐Up of Nivolumab‐Plus‐Ipilimumab in Japanese Patients With Advanced or Metastatic Renal Cell Carcinoma: A Final Analysis of the J‐ENCORE Study

**DOI:** 10.1111/iju.70400

**Published:** 2026-03-17

**Authors:** Shuzo Hamamoto, Masahiro Nozawa, Suguru Shirotake, Tomokazu Sazuka, Kazuyuki Numakura, Atsushi Mizokami, Tsunenori Kondo, Sei Naito, Takashige Abe, Kojiro Ohba, Go Kimura, Masayoshi Nagata, Shunta Onodera, Katsumi Yamaguchi, Hirotsugu Uemura

**Affiliations:** ^1^ Department of Nephro‐Urology Nagoya City University Graduate School of Medical Sciences Nagoya Japan; ^2^ Department of Urology Kindai University Faculty of Medicine Sakai Japan; ^3^ Department of Urology National Hospital Organization Osaka Minami Medical Center Kawachinagano Japan; ^4^ Department of Uro‐Oncology Saitama Medical University International Medical Center Hidaka Japan; ^5^ Department of Urology Graduate School of Medicine, Chiba University Chiba Japan; ^6^ Department of Urology Akita University Graduate School of Medicine Akita Japan; ^7^ Department of Renal and Urologic Surgery Asahikawa Medical University Asahikawa Japan; ^8^ Department of Integrative Cancer Therapy and Urology Graduate School of Medical Science, Kanazawa University Kanazawa Japan; ^9^ Department of Urology Tokyo Women's Medical University Adachi Medical Center Tokyo Japan; ^10^ Department of Urology Yamagata University Faculty of Medicine Yamagata Japan; ^11^ Department of Renal and Genitourinary Surgery Hokkaido University Graduate School of Medicine Sapporo Japan; ^12^ Department of Urology Nagasaki University Graduate School of Biomedical Sciences Nagasaki Japan; ^13^ Department of Urology Nippon Medical School Hospital Tokyo Japan; ^14^ Department of Urology Juntendo University Hospital Tokyo Japan; ^15^ Ono Pharmaceutical Co., Ltd. Osaka Japan; ^16^ Bristol Myers Squibb Tokyo Japan; ^17^ Department of Innovative Medicine Kindai University Faculty of Medicine Sakai Japan

**Keywords:** ipilimumab, Japan, nivolumab, prospective study, renal cell carcinoma

## Abstract

**Objectives:**

The J‐ENCORE study is a Japanese multicenter, prospective observational study involving patients with advanced or metastatic renal cell carcinoma treated with nivolumab‐plus‐ipilimumab combination as first‐line treatment. Interim 1‐ and 2‐year reports demonstrated efficacy and safety comparable to those of the CheckMate 214 trial, with sustained effects after nivolumab‐plus‐ipilimumab discontinuation, and explored outcome‐associated factors. This final analysis of a minimum 3‐year observation summarized long‐term outcomes, including real‐world second progression‐free survival and overall survival, and explored outcome‐associated factors.

**Methods:**

Real‐world objective response rate, response duration, real‐world progression‐free survival, overall survival, treatment‐related adverse events, and real‐world second progression‐free survival were evaluated. Outcome‐associated factors were explored.

**Results:**

The study included 274 patients (68.2% aged ≥ 65 years; 42.0%, poor risk) from 37 sites, with a median follow‐up of 47.4 (range, 36.5–59.0) months. Real‐world objective response rate was 38.4%, with 30.5% maintaining ≥ 36 months response duration. Median real‐world progression‐free survival and second progression‐free survival were 9.7 and 30.1 months, respectively, and 60.2% of patients survived for ≥ 36 months. Treatment‐related adverse events of any grade, grade 3/4, and grade 5 occurred in 78.5%, 43.1%, and 1.1% of patients, respectively. No new treatment‐related adverse events or increased frequencies were reported since the previous interim analyses. Multivariable analyses identified associations between overall survival and age, lactate dehydrogenase levels, and C‐reactive protein levels, with favorable prognosis in patients with none or one of these factors.

**Conclusions:**

We demonstrated long‐term real‐world outcomes of nivolumab‐plus‐ipilimumab. Our findings support prescriptions of nivolumab‐plus‐ipilimumab in real‐world clinical practice.

**Trail Registration:**

UMIN Clinical Trials Registry: UMIN000036772 and ClinicalTrials.gov: NCT04043975

**Abbreviations**
a/mRCC: advanced or metastatic renal cell carcinoma
AE: adverse event
ccRCC: clear cell RCC
CI: confidence interval
CRP: C‐reactive protein
DOR: duration of response
ECOG PS: Eastern Cooperative Oncology Group performance status
I/P: intermediate or poor
IMDC: International Metastatic Renal Cell Carcinoma Database Consortium
IO: immuno‐oncology
LDH: lactate dehydrogenase
MLR: monocyte‐to‐lymphocyte ratio
NIVO + IPI: nivolumab‐plus‐ipilimumab
NLR: neutrophil‐to‐lymphocyte ratio
OS: overall survival
PD: progressive disease
PFS: progression‐free survival
PLR: platelet‐to‐lymphocyte
RCC: renal cell carcinoma
RECIST: Response Evaluation Criteria in Solid Tumors
rwDOR: real‐world duration of response
rwORR: real‐world objective response rate
rwPFS: real‐world progression‐free survival
rwPFS2: real‐world second progression‐free survival
SII: systemic immune‐inflammation index
TRAEs: treatment‐related adverse events
ULN: upper limit of normal

## 1 Introduction

Over the past decade, dual immuno‐oncology (IO) combination therapy using nivolumab‐plus‐ipilimumab (NIVO + IPI) has considerably improved outcomes in advanced or metastatic renal cell carcinoma (a/mRCC) [1]. This regimen is recommended as the first‐line treatment for patients with advanced or metastatic clear cell RCC (ccRCC) classified as intermediate or poor (I/P) risk according to the International Metastatic Renal Cell Carcinoma Database Consortium (IMDC) classification [2, 3, 4]. In Japan, NIVO + IPI was approved in August 2018 for patients with previously untreated a/mRCC classified as IMDC‐based I/P risk, based on the results of the global phase 3, CheckMate 214 trial [1].

The CheckMate 214 trial excluded patients with non‐clear cell RCC, an Eastern Cooperative Oncology Group performance status (ECOG PS) ≥ 2, or brain metastases [1]. Therefore, additional real‐world evidence in Japan is needed to better understand the treatment outcomes in these patients. Additionally, IO combination therapies have demonstrated long‐term survival benefits, as evidenced by the 8‐year follow‐up results of the CheckMate 214 trial, with a median duration of response (DOR) of 82.8 months and overall survival (OS) of 46.7 months [5]. Thus, evaluating the long‐term outcomes of NIVO + IPI in diverse patient groups in real‐world clinical settings is necessary. However, most real‐world studies on NIVO + IPI have been conducted retrospectively, with short follow‐up periods and/or small sample sizes [6, 7, 8, 9, 10, 11]. Furthermore, although some patients benefit from long‐term survival, the baseline characteristics associated with favorable outcomes remain unclear. Identifying these characteristics is essential for guiding physicians' treatment decisions regarding NIVO + IPI combination therapy for each patient.

To address these gaps in real‐world evidence, we conducted a multicenter, prospective observational study, J‐ENCORE, to examine the effectiveness and safety of NIVO + IPI in patients with previously untreated a/mRCC in Japan. The first interim analysis with at least 12 months of follow‐up [12] clarified the real‐world treatment practices of NIVO + IPI and the effectiveness and safety profiles, which were comparable to those of the CheckMate 214 trial. Additionally, patient characteristics were evaluated across subgroups based on response and early progression. The second interim analysis with at least 24 months of follow‐up [13] confirmed the following: effectiveness and safety profiles comparable to those of the CheckMate 214 trial, sustained effects of NIVO + IPI even after treatment discontinuation, and occurrence of late‐onset treatment‐related adverse events (TRAEs).

In the final analysis of the J‐ENCORE study, the follow‐up period was extended to a minimum of 36 months to demonstrate the long‐term effectiveness and safety of NIVO + IPI and to investigate the discontinuation status of NIVO + IPI, including second‐line treatment and real‐world second progression‐free survival (rwPFS2), defined as the time from NIVO + IPI initiation to disease progression on second‐line treatment or death. Additionally, we investigated factors associated with OS, real‐world progression‐free survival (rwPFS), and real‐world objective response rate (rwORR) and examined the impact of the number and combination of these factors on outcomes.

## 2 Methods

### 2.1 Study Design and Patients

The J‐ENCORE study is a prospective, non‐interventional, multicenter, observational study that assessed the outcomes of NIVO + IPI in previously untreated patients with a/mRCC in real‐world settings with data collected from the medical charts of 37 Japanese medical institutions (Table S1) [12]. Patients with untreated RCC who were scheduled to initiate NIVO + IPI as first‐line treatment between August 15, 2019 and August 14, 2021, and provided written informed consent were enrolled (Figure S1). The J‐ENCORE study included patients (1) aged ≥ 20 years, (2) with histologically confirmed RCC, (3) with IMDC‐based I/P risk of RCC, (4) without prior systemic therapy for RCC, and (5) scheduled to initiate NIVO + IPI. Pregnant and/or lactating women were excluded from this study (Figure S1). Data on patient demographics, clinical status, treatment, outcomes, and adverse events (AEs) were collected from the medical records between August 2019 and August 2024. Prior to the final analysis, these data underwent a rigorous cleaning process, including logical consistency checks, to ensure data integrity and accuracy. The J‐ENCORE study was approved by the institutional review board/independent ethics committee of each study site and was registered with the UMIN Clinical Trials Registry (UMIN000036772) and ClinicalTrials.gov (NCT04043975).

### 2.2 Outcome Measures

The primary outcome was rwORR. The secondary outcomes included real‐world duration of response (rwDOR), rwPFS, OS, and incidence of AEs, and the exploratory outcomes included rwPFS2—defined as the time from initiation of NIVO + IPI to disease progression on second‐line treatment or death—and predictive biomarkers (rwORR, rwPFS, and OS) (Figure S1). Changes in tumor response were assessed by investigators using the Response Evaluation Criteria in Solid Tumors (RECIST) v1.1, based on images obtained in clinical practice. Additionally, progressive disease (PD) was identified through clinical progression. The severity of AE was assessed according to the Common Terminology Criteria for Adverse Events v4.0, Japanese Clinical Oncology Group version.

### 2.3 Statistical Analysis

Continuous variables are expressed as medians along with ranges and/or interquartile ranges, whereas categorical variables are shown as numbers with their corresponding percentages. The rwORR was determined with 95% confidence intervals (CIs) using the Clopper–Pearson method. The median rwPFS, OS, and rwDOR were estimated using the Kaplan–Meier method, with 95% CIs calculated using the Brookmeyer–Crowley method. Univariable and multivariable logistic regression analyses were conducted to identify factors associated with rwORR, rwPFS, and OS. Two‐sided *p* values < 0.05 were considered statistically significant. Statistical analyses were performed using SAS v9.4 (SAS Institute Inc., Cary, NC, USA).

## 3 Results

### 3.1 Baseline Characteristics and Patient Disposition

Overall, 274 patients who met the eligibility criteria for the J‐ENCORE study were included in the final analysis. The median follow‐up period was 47.4 (range, 36.5–59.0) months (Figure S1). Of these patients, 78.8% were male; the median age was 68 (range, 31–87) years; 68.2% were ≥ 65 years; 15.7% had ECOG PS ≥ 2; 81.8% had ccRCC; 58.0% were classified as IMDC‐based intermediate risk and 42.0% as poor risk; 50.4% had undergone previous nephrectomy; and 2.6% had brain metastases (Table 1). Of the 274 patients, 249 (90.9%) discontinued NIVO + IPI (Table S1). The reasons for discontinuation included PD in 118 patients (47.4%) and AE in 89 patients (35.7%). Subsequent therapy was administered to 136 patients (49.6%). Among these, cabozantinib was the most commonly prescribed drug in 93 patients (68.4%), followed by axitinib in 33 patients (24.3%).

**TABLE 1:** Baseline characteristics.

Characteristics	Overall, *n* = 274
Male, *n* (%)	216 (78.8)
Median age, years (range)	68 (31–87)
≥ 65 years, *n* (%)	187 (68.2)
BMI^a^, kg/m^2^, *n* (%)	
≥ 25.0	73 (26.9)
ECOG PS, *n* (%)	
0	193 (70.4)
1	38 (13.9)
2	30 (10.9)
≥ 3	13 (4.7)
Histology, *n* (%)	
ccRCC	224 (81.8)
nccRCC	50 (18.2)
IMDC risk, *n* (%)	
Intermediate	159 (58.0)
Poor	115 (42.0)
Sum of reference diameters of target lesions, mm	
*n*	250
Median (IQR)	83.4 (42.0–138.0)
With primary tumor, *n* (%)	138 (50.4)
With sarcoma component, *n* (%)	32 (11.7)
With IVC thrombosis, *n* (%)	43 (15.7)
Previous nephrectomy, *n* (%)	138 (50.4)
Site of metastasis, *n* (%)	
Lung	171 (62.4)
Lymph node	119 (43.4)
Bone	82 (29.9)
Liver	37 (13.5)
Brain	7 (2.6)
LDH ≥ 1.5 × ULN	21 (7.7)
CRP^b^, *n* (%)	
< 1 mg/dL	140 (51.3)
≥ 1 mg/dL	133 (48.7)
NLR, *n* (%)	
< 3	110 (40.1)
≥ 3	164 (59.9)
MLR, *n* (%)	
< 0.3	114 (41.6)
≥ 0.3	160 (58.4)
PLR, *n* (%)	
< 160	80 (29.2)
≥ 160	194 (70.8)
SII, *n* (%)	
< 93.7	137 (50.0)
≥ 93.7	137 (50.0)

Abbreviations: BMI, body mass index; ccRCC, clear cell renal cell carcinoma; CRP, C‐reactive protein; ECOG PS, Eastern Cooperative Oncology Group performance status; IMDC, International Metastatic Renal Cell Carcinoma Database Consortium; IQR, interquartile range; IVC, inferior vena cava; LDH, lactate dehydrogenase; MLR, monocyte‐to‐lymphocyte ratio; nccRCC, non‐clear cell renal cell carcinoma; NLR, neutrophil‐to‐lymphocyte ratio; PLR, platelet‐to‐lymphocyte ratio; SII, systemic immune‐inflammation index; ULN, upper limit of normal.

^a^ Data was missing for three patients.

^b^ Data was missing for one patient.

### 3.2 Effectiveness

**FIGURE 1:**
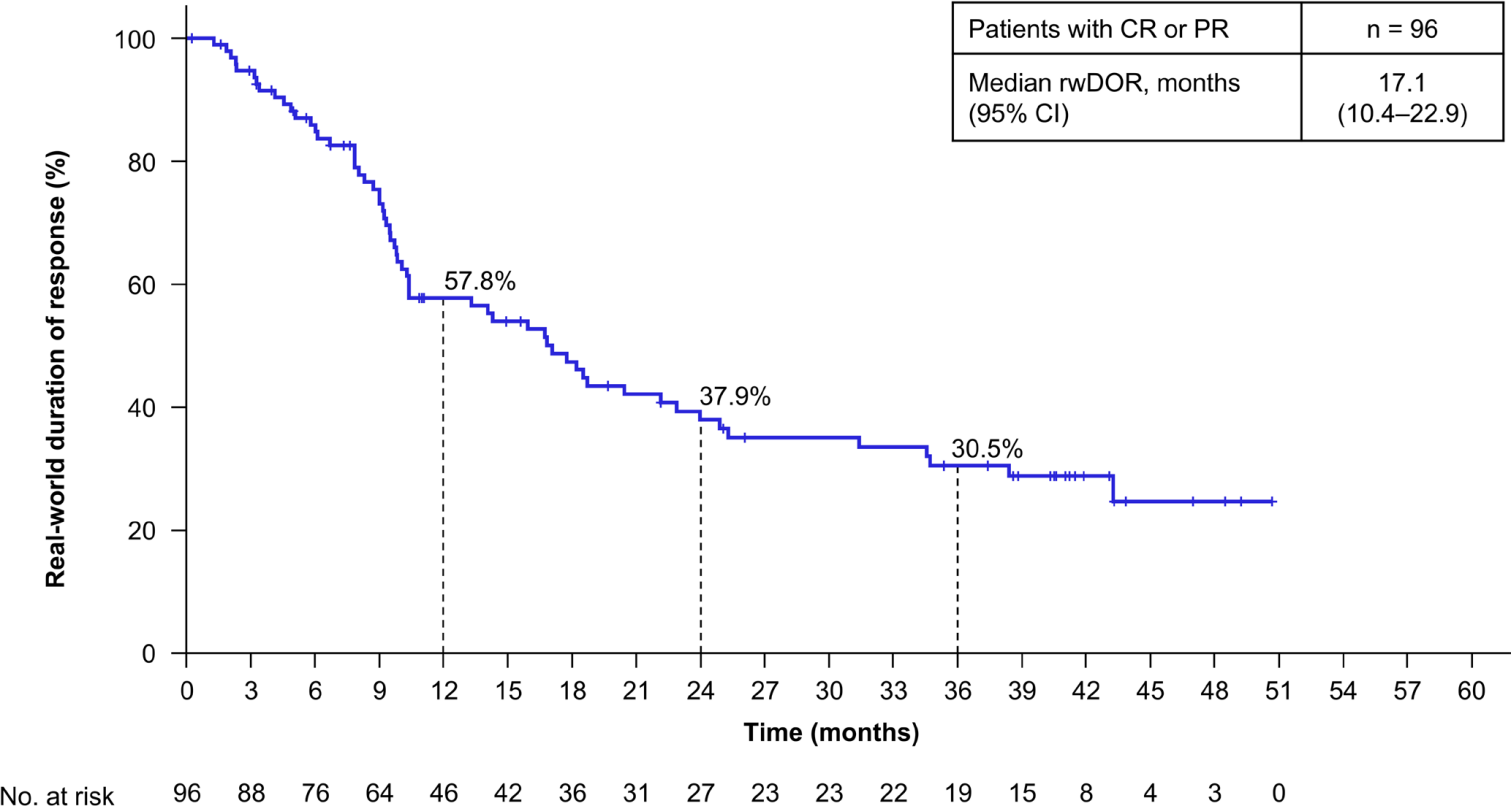
Real‐world duration of response. The rwDOR is shown for patients who achieved CR or PR as the best response. rwDOR was defined as the time from the date of the first documented CR or PR following the initiation of NIVO + IPI to the date of the first documented PD or death from any cause. Tumor response was assessed by the investigators according to the RECIST v1.1 methodology using scan images obtained in clinical practice. Additionally, clinical progression was categorized as PD. Patients without a record of PD or death were censored at the date of initiation of second‐line treatment, date of surgery, or last date of progression‐free confirmation, whichever came first. CI, confidence interval; CR, complete response; NIVO + IPI, nivolumab‐plus‐ipilimumab; PD, progressive disease; PR, partial response; RECIST, Response Evaluation Criteria in Solid Tumors; rwDOR, real‐world duration of response.

**FIGURE 2:**
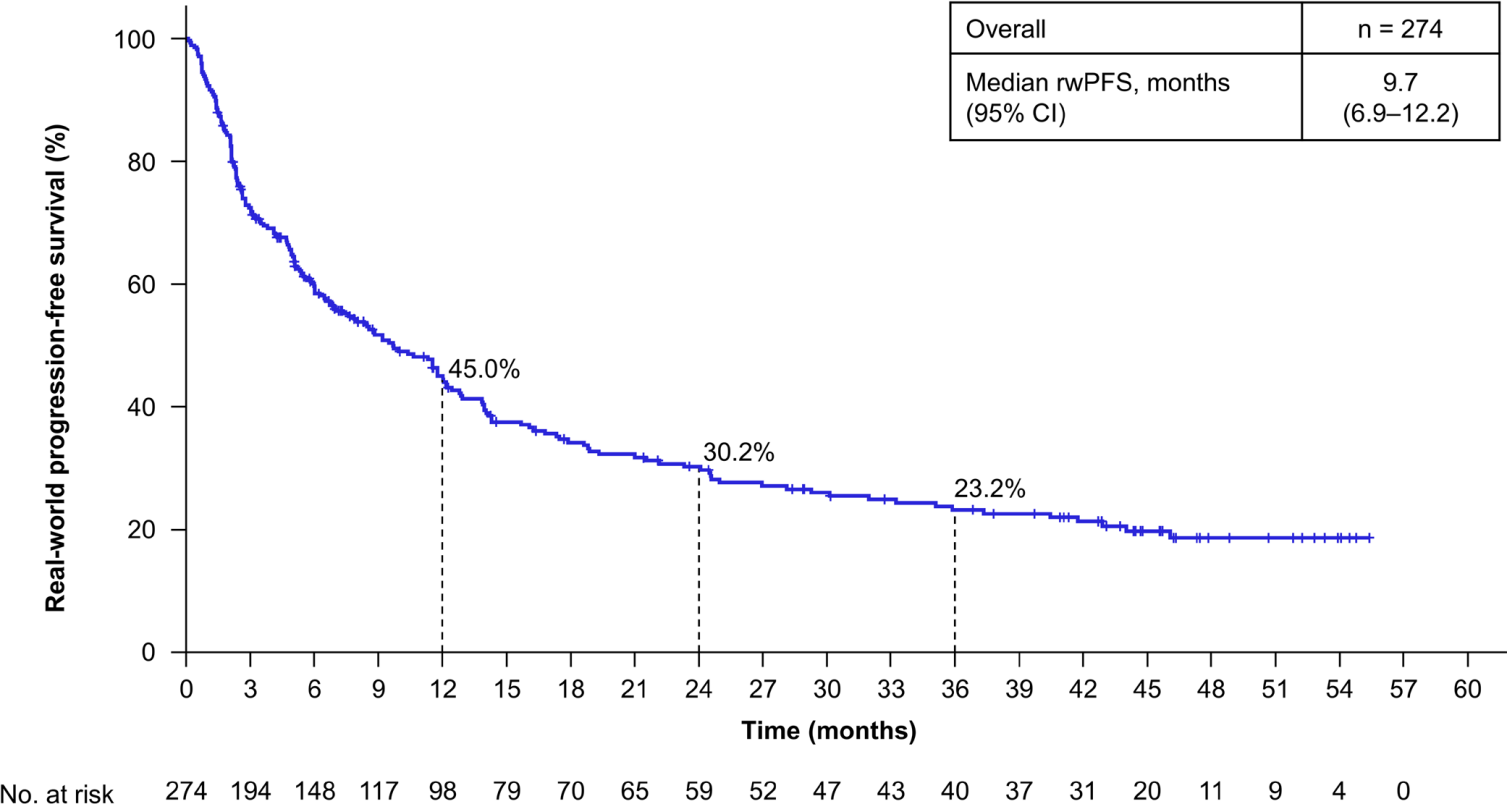
Real‐world progression‐free survival. rwPFS was defined as the time from the date of initiation of NIVO + IPI to the date of the first documented PD or death from any cause. Tumor response was assessed by the investigators according to the RECIST v1.1 methodology using scan images obtained in clinical practice. Additionally, clinical progression was categorized as PD. Patients without a record of PD or death were censored at the date of initiation of second‐line treatment, date of surgery, or last date of progression‐free confirmation, whichever came first. CI, confidence interval; NIVO + IPI, nivolumab‐plus‐ipilimumab; PD, progressive disease; RECIST, Response Evaluation Criteria in Solid Tumors; rwPFS, real‐world progression‐free survival.

**FIGURE 3:**
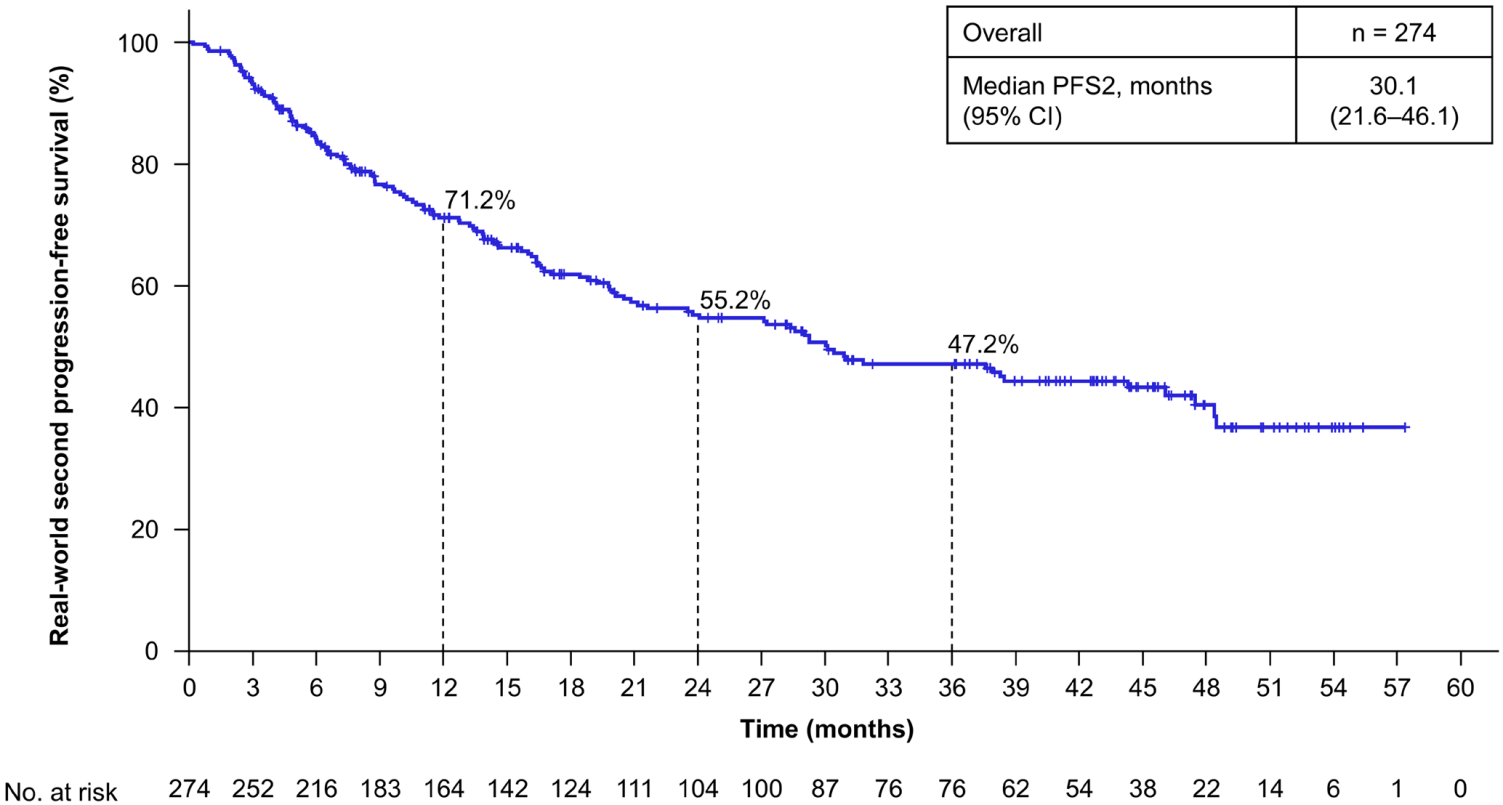
Real‐world second progression‐free survival. rwPFS2 was defined as the time from the date of initiation of NIVO + IPI to the date of the first documented PD on second‐line treatment or death from any cause. Tumor response was assessed by the investigators according to the RECIST v1.1 methodology using scan images obtained in clinical practice. Additionally, clinical progression was categorized as PD. Patients without a record of PD on second‐line treatment or death were censored at the date of initiating third‐line treatment, date of surgery, or last date of progression‐free confirmation, whichever came first. CI, confidence interval; NIVO + IPI, nivolumab‐plus‐ipilimumab; PD, progressive disease; RECIST, Response Evaluation Criteria in Solid Tumors; rwPFS2, real‐world second progression‐free survival.

**FIGURE 4:**
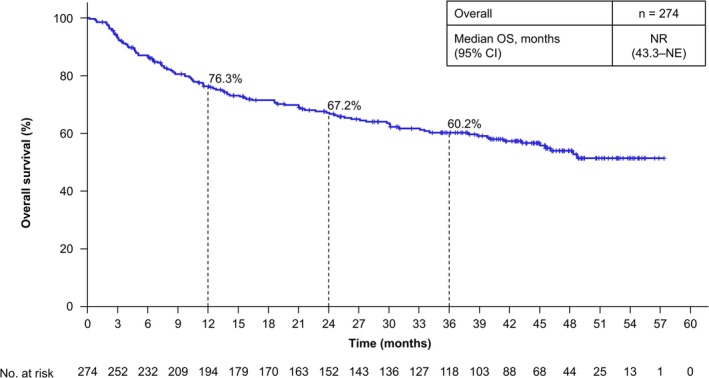
Overall survival. OS was defined as the time from the date of initiation of NIVO + IPI to the date of death from any cause. CI, confidence interval; NE, not estimated; NIVO + IPI, nivolumab‐plus‐ipilimumab; NR, not reached; OS, overall survival.

The rwORR for patients with measurable disease was 38.4% (95% CI, 32.3–44.7) (Table 2). The median rwDOR of patients with an objective response of complete response or partial response was 17.1 months (95% CI, 10.4–22.9) (Figure 1). Among these responders, 30.5% maintained response for ≥ 36 months. The median rwPFS of all patients who initiated NIVO + IPI was 9.7 months (95% CI, 6.9–12.2), with a 36‐month rwPFS rate of 23.2% (Figure 2). The median rwPFS2 of all patients who initiated NIVO + IPI was 30.1 months (95% CI, 21.6–46.1), with a 36‐month rwPFS2 rate of 47.2% (Figure 3). The median OS of all patients who initiated NIVO + IPI was not reached (95% CI, 43.3 months–not estimated), with a 36‐month OS rate of 60.2% (Figure 4).

**TABLE 2:** Real‐world objective response rate.

	Overall, *n* = 274
With measurable disease^a^, *n*	250
rwORR, % (95% CI)	38.4 (32.3–44.7)
BOR, *n* (%)	
CR	17 (6.8)
PR	79 (31.6)
SD	70 (28.0)
PD	59 (23.6)
NE^b^	25 (10.0)
DCR, % (95% CI)	66.4 (60.2–72.2)

Abbreviations: BOR, best overall response; CI, confidence interval; CR, complete response; DCR, disease control rate; NE, not evaluable; PD, progressive disease; PR, partial response; RECIST, Response Evaluation Criteria in Solid Tumors; rwORR, real‐world objective response rate; SD, stable disease.

^a^ Measurable disease was assessed by the investigators per RECIST v1.1.

^b^ NE includes 23 patients whose disease assessment was not conducted.

### 3.3 Safety

Among the 274 patients who initiated NIVO + IPI, TRAEs of any grade occurred in 215 patients (78.5%), grade 3/4 TRAEs in 118 (43.1%), and grade 5 TRAEs in 3 (1.1%) (Table 3). The most frequent TRAEs of any grade were skin‐related events in 102 patients (37.2%), followed by endocrine‐related events in 86 patients (31.4%). Three patients (1.1%) died from grade 5 TRAEs of the liver, gastrointestinal system, and cardiovascular system. During the extended follow‐up of ≥ 3 years, no new TRAEs or elevations in their frequencies were reported, as compared to the findings of the previous 2‐year interim analysis [13].

**TABLE 3:** Treatment‐related adverse events.

*n* (%)	Overall, *n* = 274		
	Any grade	Grade 3/4	Grade 5
TRAEs	215 (78.5)	118 (43.1)	3 (1.1)
Skin	102 (37.2)	11 (4.0)	0 (0)
Endocrine	86 (31.4)	30 (10.9)	0 (0)
Hepatic	44 (16.1)	25 (9.1)	1 (0.4)
Respiratory	24 (8.8)	9 (3.3)	0 (0)
Diarrhea/colitis	22 (8.0)	11 (4.0)	0 (0)
Metabolism	18 (6.6)	16 (5.8)	0 (0)
Renal	15 (5.5)	8 (2.9)	0 (0)
Musculoskeletal	15 (5.5)	4 (1.5)	0 (0)
Gastrointestinal	11 (4.0)	6 (2.2)	1 (0.4)
Cardiovascular	11 (4.0)	5 (1.8)	1 (0.4)
Nervous	11 (4.0)	5 (1.8)	0 (0)
Hematotoxicity	8 (2.9)	8 (2.9)	0 (0)
Acute pancreatitis	4 (1.5)	3 (1.1)	0 (0)
Increased amylase and lipase levels	3 (1.1)	3 (1.1)	0 (0)
Other	58 (21.2)	14 (5.1)	0 (0)

*Note:* Grade was assessed by the investigators according to CTCAE v4.0.

Abbreviations: CTCAE, Common Terminology Criteria for Adverse Events; TRAE, treatment‐related adverse event.

### 3.4 Outcome‐Associated Factors

Regarding factors associated with rwORR, univariable analysis identified bone metastasis as the only variable associated with rwORR; therefore, multivariable analysis was not performed (Table 4).

**TABLE 4:** Univariable analysis of baseline characteristics associated with real‐world objective response rate.

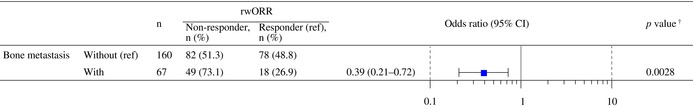

*Note:* Univariable analysis was conducted to identify variables associated with rwORR. Responders denote patients whose BOR was CR or PR, and non‐responders denote patients whose BOR was PD or SD, as determined by the investigators per RECIST v1.1. Univariable analysis identified bone metastasis as the only variable associated with rwORR (*p* < 0.05); therefore, multivariable analysis was not conducted.

Abbreviations: BOR, best overall response; CI, confidence interval; CR, complete response; PD, progressive disease; PR, partial response; RECIST, Response Evaluation Criteria in Solid Tumors; ref., reference; rwORR, real‐world objective response rate; SD, stable disease.

^†^ The *p* values were calculated based on maximum likelihood estimation.

Regarding factors associated with rwPFS, univariable analysis identified several variables, including IMDC‐based risk, primary tumor, inferior vena cava thrombosis, liver metastasis, lactate dehydrogenase (LDH) levels, C‐reactive protein (CRP) levels, monocyte‐to‐lymphocyte ratio (MLR), platelet‐to‐lymphocyte ratio (PLR), and systemic immune‐inflammation index (SII). Among these variables, multivariable analysis identified IMDC‐based poor risk and CRP levels ≥ 1 mg/dL as baseline risk factors associated with shorter rwPFS (Table 5). Figure S2 shows the rwPFS stratified by patient subgroups according to the number of baseline risk factors present (0, 1, or 2). Patients with one or two baseline risk factors exhibited a significantly shorter median rwPFS than did those without. The 36‐month rwPFS rates for patients with 0, 1, and 2 factors were 37.3%, 13.2%, and 9.2%, respectively. Figure S3A,B shows the rwPFS for each baseline risk factor (CRP levels and IMDC‐based risk), with the rwPFS for combining these two factors shown in Figure S3C.

**TABLE 5:** Multivariable analysis of baseline characteristics associated with real‐world progression‐free survival.

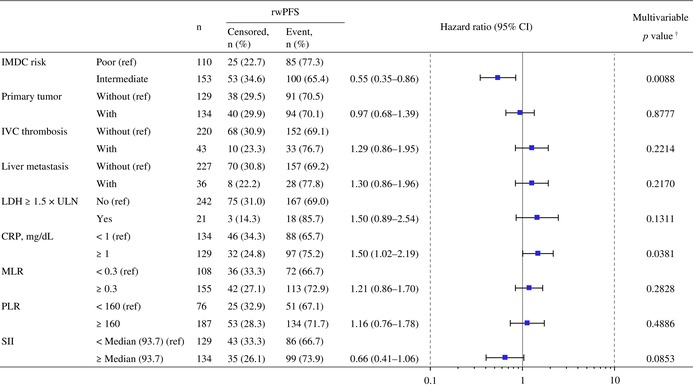

*Note:* Multivariable analysis was conducted using variables selected based on the results of the univariable analysis (*p* < 0.05) to identify baseline risk factors associated with rwPFS. Event of rwPFS was defined as the first documented PD or death on NIVO + IPI combination therapy. Censored cases included patients who initiated second‐line therapy, underwent surgery, or were alive without documented PD at the last follow‐up date.

Abbreviations: CI, confidence interval; CRP, C‐reactive protein; IMDC, International Metastatic Renal Cell Carcinoma Database Consortium; IVC, inferior vena cava; LDH, lactate dehydrogenase; MLR, monocyte‐to‐lymphocyte ratio; NIVO + IPI, nivolumab‐plus‐ipilimumab; PD, progressive disease; PLR, platelet‐to‐lymphocyte ratio; ref., reference; rwPFS, real‐world progression‐free survival; SII, systemic immune‐inflammation index; ULN, upper limit of normal.

^†^ The *p* values were calculated based on maximum likelihood estimation.

**FIGURE 5:**
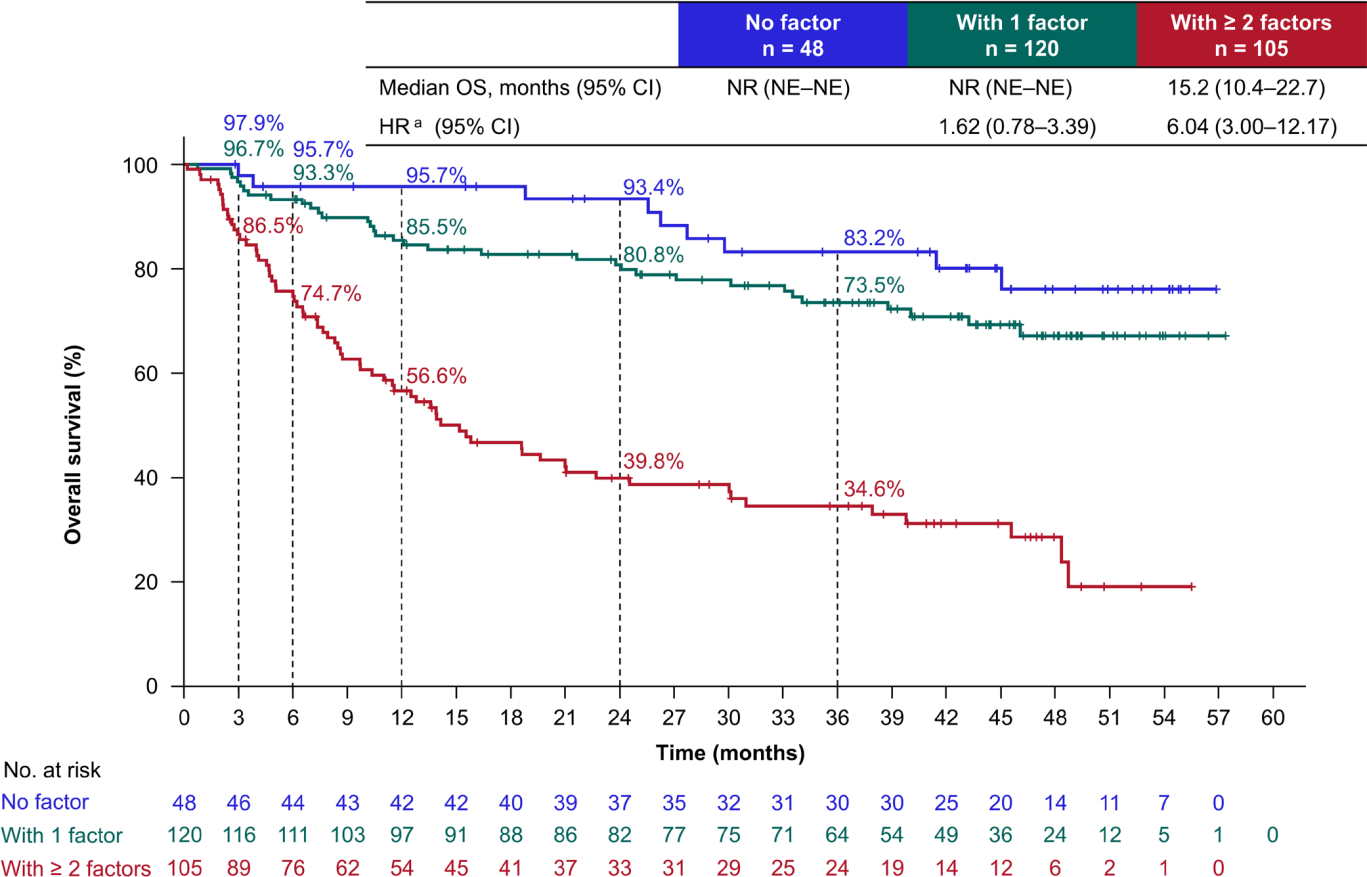
Overall survival stratified by patient subgroups according to the number of baseline risk factors. OS was analyzed by patient subgroups according to the number of baseline risk factors. Age ≥ 65 years, LDH levels ≥ 1.5 × ULN, and CRP levels ≥ 1 mg/dL were identified as baseline risk factors associated with OS through multivariable analysis, as shown in Table 6. For example, in the case of a patient (72 years old, LDH levels ≥ 1.5 × ULN, CRP 0.9 mg/dL), the patient would be classified as with ≥ 2 factors. OS was defined as the time from the date of initiation of NIVO + IPI to the date of death from any cause. ^a^HR was calculated using the subgroup with no factor as a reference. CI, confidence interval; CRP, C‐reactive protein; HR, hazard ratio; LDH, lactate dehydrogenase; NE, not estimated; NIVO + IPI, nivolumab‐plus‐ipilimumab; NR, not reached; OS, overall survival; ULN, upper limit of normal.

Regarding factors associated with OS, univariable analysis identified several variables, including age, body mass index, histology, IMDC‐based risk, primary tumor, lymph node metastasis, liver metastasis, LDH levels, CRP levels, neutrophil‐to‐lymphocyte ratio (NLR), MLR, PLR, and SII. Among these variables, multivariable analysis identified age (≥ 65 years), LDH levels ≥ 1.5 × upper limit of normal (ULN), and CRP levels ≥ 1 mg/dL as baseline risk factors associated with shorter OS (Table 6). The OS stratified by patient subgroups according to the number of identified baseline risk factors present (0, 1, or ≥ 2) is shown in Figure 5. The 36‐month OS rates for patients with 0, 1, and ≥ 2 factors were 83.2%, 73.5%, and 34.6%, respectively.

**TABLE 6:** Multivariable analysis of baseline characteristics associated with overall survival.

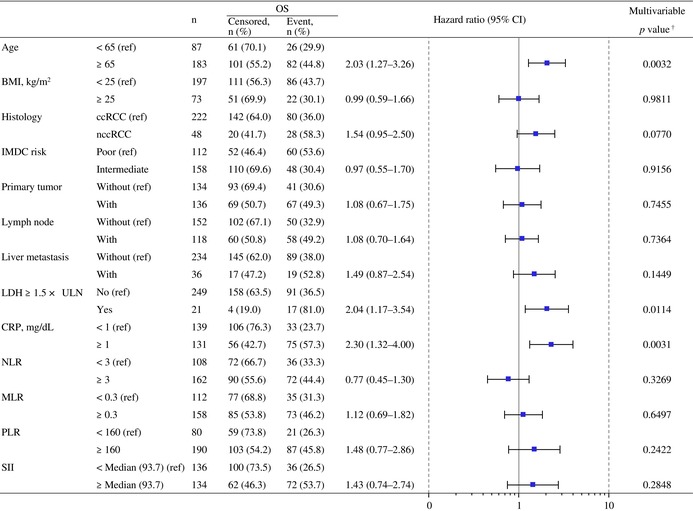

*Note:* Multivariable analysis was conducted using variables selected based on the results of the univariable analysis (*p* < 0.05) to identify baseline risk factors associated with OS. Event of OS was defined as death from any cause. Censored cases included patients who were alive at the last follow‐up date.

Abbreviations: BMI, body mass index; ccRCC, clear cell renal cell carcinoma; CI, confidence interval; CRP, C‐reactive protein; IMDC, International Metastatic Renal Cell Carcinoma Database Consortium; LDH, lactate dehydrogenase; MLR, monocyte‐to‐lymphocyte ratio; nccRCC, non‐clear cell renal cell carcinoma; NLR, neutrophil‐to lymphocyte ratio; OS, overall survival; PLR, platelet‐to‐lymphocyte ratio; ref., reference; SII, systemic immune‐inflammation index; ULN, upper limit of normal.

^†^ The *p* values were calculated based on maximum likelihood estimation.

The OS stratified by patient subgroups for each factor, including age, LDH, and CRP, is shown in Figure S4A–C. The OS stratified by patient subgroups according to combining two of the three risk factors is shown in Figure S4D–F.

## 4 Discussion

The final analysis of the J‐ENCORE study, with an extended follow‐up of 3–5 years, demonstrated the long‐term effectiveness of NIVO + IPI, consistent with the previous interim report findings with a follow‐up of at least 1 and 2 years [12, 13]. In the final analysis, the rwPFS appeared to reach a plateau at approximately 36 months. Given that the PFS in the CheckMate 214 trial plateaued after 24 months and the plateau was sustained for over 24 months, a longer observation period may be required to adequately assess the durability of effectiveness in real‐world settings [14]. The median rwDOR and rwPFS were shorter than those reported in the CheckMate 214 trial [14], likely due to variations in patients' baseline characteristics between clinical trials and real‐world settings [13]. Nonetheless, some patients in real‐world practice experience sustained antitumor effects.

Although the median rwDOR and rwPFS of the J‐ENCORE study were shorter than those of the CheckMate 214 trial [14], the median OS was comparable. OS outcomes may have been influenced by subsequent therapies. In the CheckMate 214 trial [15], 51.3% of patients transitioned to second‐line treatment, which was comparable to the 49.6% observed in the J‐ENCORE study. Notably, the patterns of second‐line treatment differed between the two studies. Specifically, in the CheckMate 214 trial, the most common drug for second‐line treatment was sunitinib (23.8%), followed by pazopanib (18.4%) and axitinib (17.9%), whereas in the J‐ENCORE study, most patients received cabozantinib (68.4%), followed by axitinib (24.3%). Cabozantinib demonstrated longer PFS than sunitinib [16], suggesting that novel tyrosine kinase inhibitors, such as cabozantinib, as second‐line treatment could have contributed to the favorable OS in the J‐ENCORE study. Thus, differences in the patterns of second‐line treatments may explain comparable OS despite differences in DOR and PFS in both studies.

The median rwPFS2 was 30.1 months, which exceeds approximately three times the median rwPFS of 9.7 months. In Japanese patients in the CheckMate 214 trial, the objective response rate for second‐line treatment was 32%, and the disease control rate was 84%, indicating favorable outcomes [17]. The favorable rwPFS2 reflects effective disease control achieved through second‐line treatment following NIVO + IPI in most patients, including those who experienced PD after treatment. This suggests that even if first‐line treatment with NIVO + IPI is unsuccessful, second‐line treatment may yield positive outcomes. Overall, our findings demonstrated that NIVO + IPI is effective in achieving sustained antitumor responses and long‐term survival in Japanese patients with a/mRCC in real‐world clinical settings.

The safety profiles of NIVO + IPI remained consistent with those of previous reports [6, 7, 8, 9, 10, 11, 12, 13, 15], with no new TRAEs or increases in their frequencies observed during extended follow‐up, confirming manageable safety over prolonged durations.

Univariable analysis for baseline characteristics identified bone metastases as the only variable associated with lower rwORR. Multivariable analysis revealed that CRP ≥ 1 mg/dL and IMDC‐based poor risk were associated with shorter rwPFS, and CRP ≥ 1 mg/dL, LDH ≥ 1.5 × ULN, and age ≥ 65 years were associated with shorter OS, consistent with those reported in previous studies [9, 18]. Furthermore, we analyzed subgroups of patients aged ≥ 65 and < 65 years. Previous studies have reported that patients aged ≥ 75 years exhibited shorter OS when treated with NIVO + IPI than those aged < 75 years [9, 19]. Conversely, another study reported favorable OS in patients aged ≥ 75 years [20]. These reports highlight the inconsistency and heterogeneity surrounding the age‐related efficacy of NIVO + IPI. In the J‐ENCORE study, although the median OS in patients aged ≥ 65 years was relatively shorter than that in those aged < 65 years, it remained favorable (median OS: 46.1 months), suggesting that NIVO + IPI can provide survival benefits for older patients with a/mRCC. Additionally, elevated CRP and LDH levels were identified as risk factors associated with OS in patients treated with NIVO + IPI. These factors are known prognostic indicators of RCC [21, 22, 23, 24, 25, 26, 27, 28], and their association with tumor burden has also been reported [21, 22, 29]. Therefore, patients with elevated CRP and LDH levels were presumed to have a high tumor burden, and regardless of treatment with NIVO + IPI, a high tumor burden may lead to poor prognosis. CRP is a known prognostic factor for OS in patients receiving NIVO + IPI [30], supporting our study findings. The CheckMate 214 trial showed an association between LDH levels and OS, with similar findings observed for sunitinib [18], suggesting that the baseline risk factors identified in the J‐ENCORE study are not exclusive to NIVO + IPI but may represent general risk factors for RCC.

Patients with no or one risk factor showed relatively favorable OS. Even with one risk factor (age, LDH levels, or CRP levels), the prognosis remained favorable, and early death was uncommon. We previously developed a nomogram using identified individual factors to predict early PD. The nomogram for early PD incorporated multiple factors, including sex, liver metastasis, history of surgery, IMDC‐based risk, CRP levels, and NLR levels [12]. Notably, CRP levels were a common factor when compared to the OS‐related factors identified in the final analysis of this study. These findings suggest that prognosis cannot be accurately predicted based on a single factor alone. Instead, a comprehensive evaluation integrating multiple factors may yield more precise prognostic predictions. Therefore, our findings provide valuable insights for clinical practice and underscore the importance of adopting a multifactorial approach to prognosis assessment.

This study had some limitations. As this was an observational study without a comparison group, assessing the relative efficacy and safety was limited. Selection bias may have existed because the enrolled patients may not represent the overall a/mRCC population. Treatment assessments and decisions were made by treating physicians without a central review. The multivariable analysis was exploratory in nature. While investigating the outcomes of the combination of baseline risk factors, some combinations had a limited sample size, potentially affecting data reliability.

In conclusion, the final analysis of the J‐ENCORE study, with a minimum follow‐up of 3 years, demonstrated long‐term effectiveness and safety of NIVO + IPI in Japanese patients with a/mRCC, consistent with the findings reported in the CheckMate 214 trial. Multivariable analysis identified age, LDH levels, and CRP levels as baseline risk factors associated with OS, with a favorable prognosis in patients with none or one of these factors. The safety profiles remained consistent with those of previous reports, with no new TRAEs or increased frequencies noted during the extended follow‐up period, underscoring the manageable safety of NIVO + IPI over a prolonged treatment period. Overall, the findings from the J‐ENCORE study provide valuable insights into the long‐term effectiveness and safety of NIVO + IPI in real‐world clinical settings, supporting its use as first‐line treatment for Japanese patients with a/mRCC.

## Author Contributions

**Shuzo Hamamoto:** conceptualization, investigation, resources, supervision, visualization, writing‐original draft, writing‐review and editing. **Masahiro Nozawa:** conceptualization, investigation, resources, writing‐review and editing. **Suguru Shirotake, Tomokazu Sazuka, Kazuyuki Numakura, Atsushi Mizokami, Tsunenori Kondo, Sei Naito, Takashige Abe, Kojiro Ohba,** and **Masayoshi Nagata:** investigation, resources, writing‐review and editing. **Go Kimura:** conceptualization, formal analysis, investigation, resources, writing‐review and editing. **Shunta Onodera** and **Katsumi Yamaguchi:** conceptualization, formal analysis, funding acquisition, methodology, project administration, resources, visualization, writing‐original draft, and writing‐review and editing. **Hirotsugu Uemura:** conceptualization, formal analysis, investigation, methodology, resources, supervision, visualization, writing‐original draft, writing‐review and editing.

## Funding

This study was funded by Bristol Myers Squibb and Ono Pharmaceutical Co. Ltd.

## Ethics Statement

The protocol for this research project has been approved by the Ethics Committee of Kindai University Hospital (Approval No. 31‐061). In addition, this study was approved by the ethics committee of all participating hospitals and conforms to the provisions of the Ethical Guidelines and the Declaration of Helsinki.

## Consent

Written informed consent was obtained from each patient before data collection.

## Conflicts of Interest

Shuzo Hamamoto received research grants or contracts from Daiwa Securities Co. Ltd., and Toyoaki Shogakukai; honoraria from Olympus Corporation, Boston Scientific Corporation, Nipro Corporation, Ono Pharmaceutical Co. Ltd., Eisai Co. Ltd., and MSD K.K.; and patent support (planned, issued, or pending) with Goodman Co. Ltd., and Create Medic Co. Ltd. Suguru Shirotake, Tomokazu Sazuka, Sei Naito, and Go Kimura received honoraria from Bristol Myers Squibb and Ono Pharmaceutical Co. Ltd. Kazuyuki Numakura received honoraria from Bristol Myers Squibb, Ono Pharmaceutical Co. Ltd., Eisai Co. Ltd., and Takeda Pharmaceutical Company Limited. Tsunenori Kondo received honoraria from Ono Pharmaceutical Co. Ltd. Masayoshi Nagata received honoraria from Astellas Pharma Inc., Bayer Yakuhin Ltd., Eisai Co. Ltd., Janssen Pharmaceutical K.K., Kyowa Kirin Co. Ltd., Merck Biopharma Co. Ltd., and Sanofi K.K. Shunta Onodera is an employee of Ono Pharmaceutical Co. Ltd. Katsumi Yamaguchi is an employee of Bristol Myers Squibb. Hirotsugu Uemura received honoraria from Bristol Myers Squibb, Ono Pharmaceutical Co. Ltd., and Takeda Pharmaceutical Company Limited and research funds from Ono Pharmaceutical Co. Ltd., Takeda Pharmaceutical Company Limited, Astellas Pharma Inc., and Pfizer Japan Inc. The other authors (Masahiro Nozawa, Atsushi Mizokami, Takashige Abe, and Kojiro Ohba) have no conflicts of interest to disclose. Kazuyuki Numakura, Atsushi Mizokami, Sei Naito, and Takashige Abe are the Editorial Board members of the International Journal of Urology and the co‐authors of this article. To minimize bias, they were excluded from all editorial decision‐making related to the acceptance of this article for publication.

## Supporting information


**Figure S1:** J‐ENCORE study design.
**Figure S2:** Real‐world progression‐free survival stratified by patient subgroups according to the number of baseline risk factors.
**Figure S3:** (A) Real‐world progression‐free survival stratified by patient subgroups according to CRP levels. (B) Real‐world progression‐free survival stratified by patient subgroups according to IMDC‐based risk. (C) Real‐world progression‐free survival stratified by patient subgroups according to two combined risk factors.
**Figure S4:** (A) Overall survival stratified by patient subgroups according to age. (B) Overall survival stratified by patient subgroups according to LDH levels. (C) Overall survival stratified by patient subgroups according to CRP levels. (D) Overall survival stratified by patient subgroups according to two combined risk factors (CRP and LDH levels). (E) Overall survival stratified by patient subgroups according to two combined risk factors (CRP levels and age). (F) Overall survival stratified by patient subgroups according to two combined risk factors (age and LDH levels).

**Table S1:** List of investigators.
**Table S2:** Summary of nivolumab‐plus‐ipilimumab discontinuation and subsequent therapy.

## Data Availability

Bristol Myers Squibb's policy on data sharing may be found at: https://www.bms.com/researchers‐and‐partners/independent‐research/data‐sharing‐request‐process.html.
