# Supplementary material for: A Minimum 3‐Year Follow‐Up of Nivolumab‐Plus‐Ipilimumab in Japanese Patients With Advanced or Metastatic Renal Cell Carcinoma: A Final Analysis of the J‐ENCORE Study
Source: Int J Urol. 2026 Mar 17;33(3):e70400. doi: 10.1111/iju.70400 (PMC12993795; doi:10.1111/iju.70400)
Supplement: Supplementary file 1 — Figure S1: J‐ENCORE study design. Figure S2: Real‐world progression‐free survival stratified by patient subgroups according to the number of baseline risk factors. Figure S3: (A) Real‐world progression‐free survival stratified by patient subgroups according to CRP levels. (B) Real‐world progression‐free survival stratified by patient subgroups according to IMDC‐based risk. (C) Real‐world progression‐free survival stratified by patient subgroups according to two combined risk factors. Figure S4: (A) Overall survival stratified by patient subgroups according to age. (B) Overall survival stratified by patient subgroups according to LDH levels. (C) Overall survival stratified by patient subgroups according to CRP levels. (D) Overall survival stratified by patient subgroups according to two combined risk factors (CRP and LDH levels). (E) Overall survival stratified by patient subgroups according to two combined risk factors (CRP levels and age). (F) Overall survival stratified by patient subgroups according to two combined risk factors (age and LDH levels). [file IJU-33-0-s002.pptx]

## Slide 1
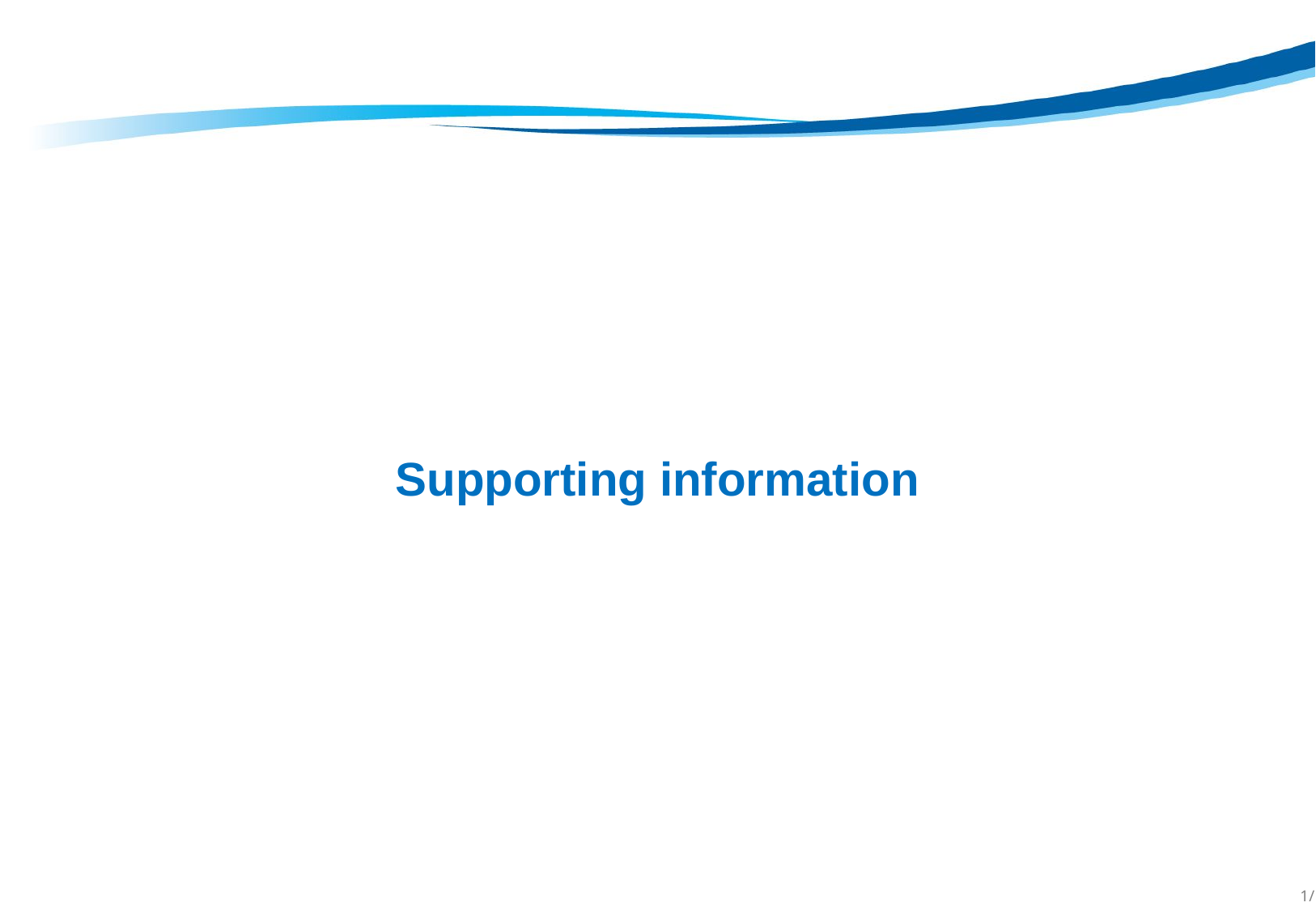

# Supporting information

## Slide 2
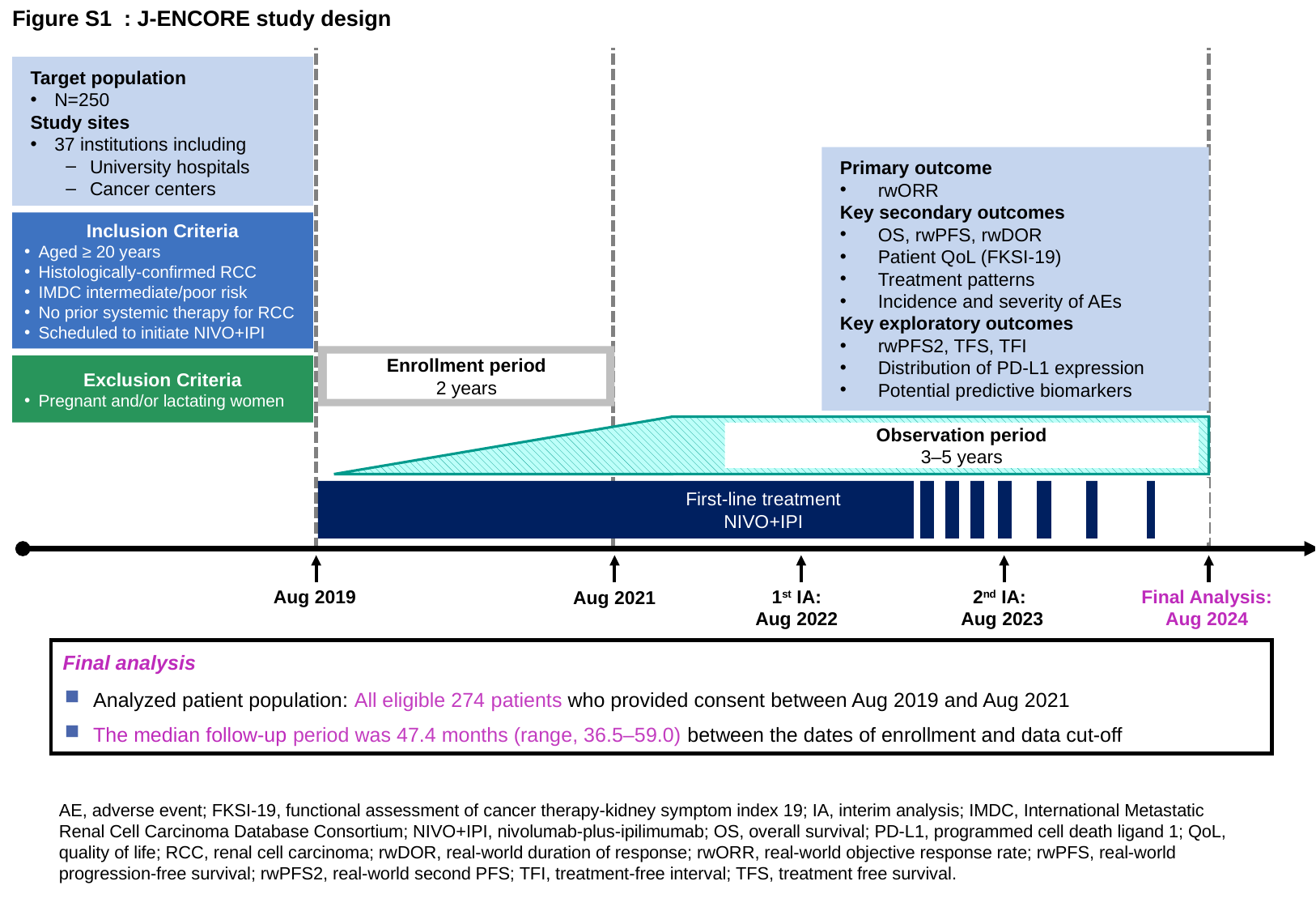

Figure S1 : J-ENCORE study design
Target population
N=250
Study sites
37 institutions including
University hospitals
Cancer centers
Primary outcome
rwORR
Key secondary outcomes
OS, rwPFS, rwDOR
Patient QoL (FKSI-19)
Treatment patterns
Incidence and severity of AEs
Key exploratory outcomes
rwPFS2, TFS, TFI
Distribution of PD-L1 expression
Potential predictive biomarkers
Inclusion Criteria
Aged ≥ 20 years
Histologically-confirmed RCC
IMDC intermediate/poor risk
No prior systemic therapy for RCC
Scheduled to initiate NIVO+IPI
Enrollment period
2 years
Exclusion Criteria
Pregnant and/or lactating women
Observation period
3–5 years
First-line treatment
NIVO+IPI
Aug 2019
1st IA:
Aug 2022
2nd IA:
Aug 2023
Final Analysis:
Aug 2024
Aug 2021
Final analysis
Analyzed patient population: All eligible 274 patients who provided consent between Aug 2019 and Aug 2021
The median follow-up period was 47.4 months (range, 36.5–59.0) between the dates of enrollment and data cut-off
AE, adverse event; FKSI-19, functional assessment of cancer therapy-kidney symptom index 19; IA, interim analysis; IMDC, International Metastatic Renal Cell Carcinoma Database Consortium; NIVO+IPI, nivolumab-plus-ipilimumab; OS, overall survival; PD-L1, programmed cell death ligand 1; QoL, quality of life; RCC, renal cell carcinoma; rwDOR, real-world duration of response; rwORR, real-world objective response rate; rwPFS, real-world progression-free survival; rwPFS2, real-world second PFS; TFI, treatment-free interval; TFS, treatment free survival.

## Slide 3
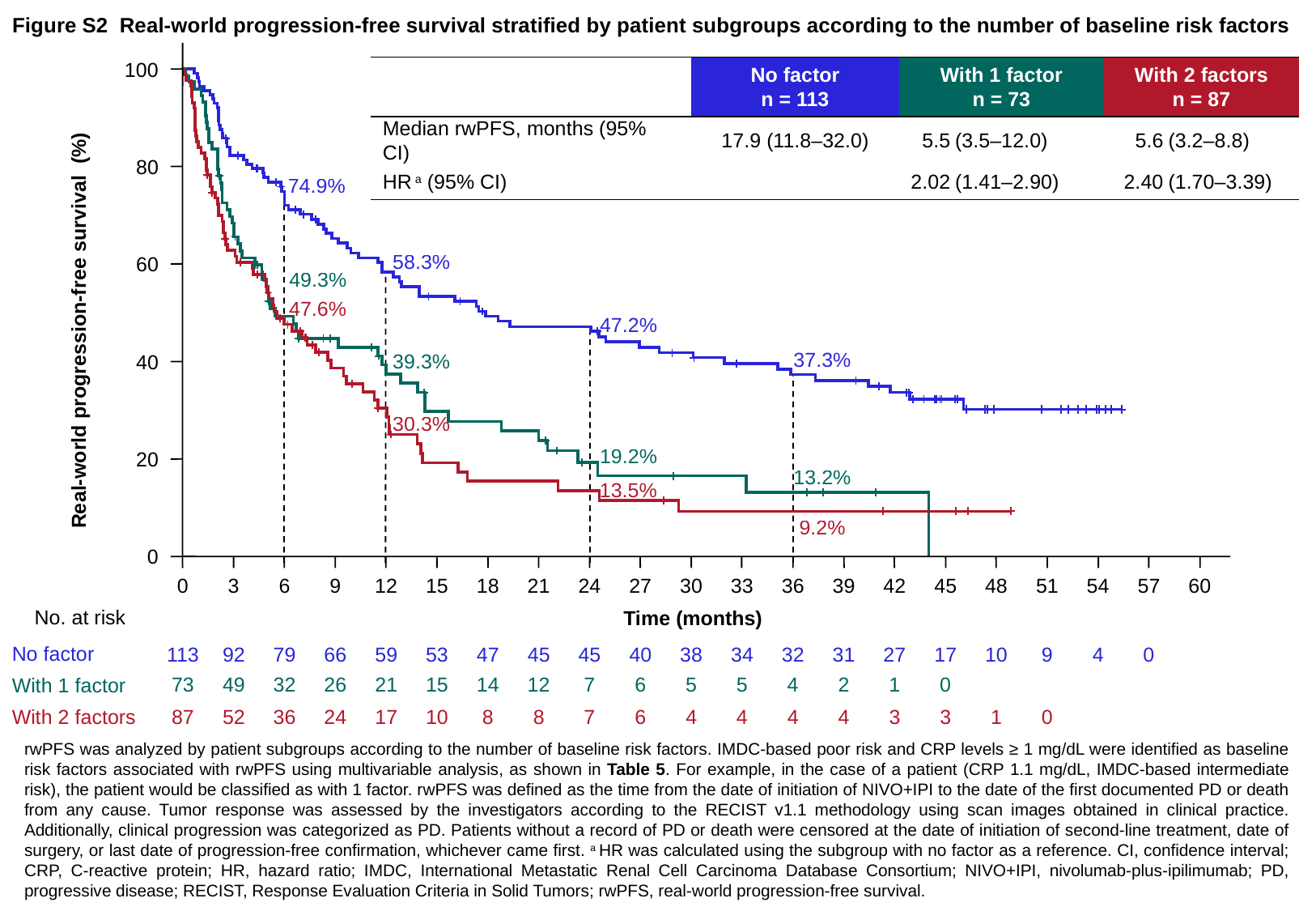

Figure S2 Real-world progression-free survival stratified by patient subgroups according to the number of baseline risk factors
100
| | No factor n = 113 | With 1 factor n = 73 | | With 2 factors n = 87 | |
| --- | --- | --- | --- | --- | --- |
| Median rwPFS, months (95% CI) | 17.9 (11.8–32.0) | 5.5 | (3.5–12.0) | 5.6 | (3.2–8.8) |
| HR a (95% CI) | | 2.02 | (1.41–2.90) | 2.40 | (1.70–3.39) |
80
74.9%
58.3%
60
49.3%
47.6%
47.2%
Real-world progression-free survival (%)
37.3%
39.3%
40
30.3%
19.2%
20
13.2%
13.5%
9.2%
0
0
3
6
9
12
15
18
21
24
27
30
33
36
39
42
45
48
51
54
57
60
No. at risk
Time (months)
No factor
113
92
79
66
59
53
47
45
45
40
38
34
32
31
27
17
10
9
4
0
With 1 factor
73
49
32
26
21
15
14
12
7
6
5
5
4
2
1
0
87
52
36
24
17
10
8
8
7
6
4
4
4
4
3
3
1
0
With 2 factors
rwPFS was analyzed by patient subgroups according to the number of baseline risk factors. IMDC-based poor risk and CRP levels ≥ 1 mg/dL were identified as baseline risk factors associated with rwPFS using multivariable analysis, as shown in Table 5. For example, in the case of a patient (CRP 1.1 mg/dL, IMDC-based intermediate risk), the patient would be classified as with 1 factor. rwPFS was defined as the time from the date of initiation of NIVO+IPI to the date of the first documented PD or death from any cause. Tumor response was assessed by the investigators according to the RECIST v1.1 methodology using scan images obtained in clinical practice. Additionally, clinical progression was categorized as PD. Patients without a record of PD or death were censored at the date of initiation of second-line treatment, date of surgery, or last date of progression-free confirmation, whichever came first. a HR was calculated using the subgroup with no factor as a reference. CI, confidence interval; CRP, C-reactive protein; HR, hazard ratio; IMDC, International Metastatic Renal Cell Carcinoma Database Consortium; NIVO+IPI, nivolumab-plus-ipilimumab; PD, progressive disease; RECIST, Response Evaluation Criteria in Solid Tumors; rwPFS, real-world progression-free survival.

## Slide 4
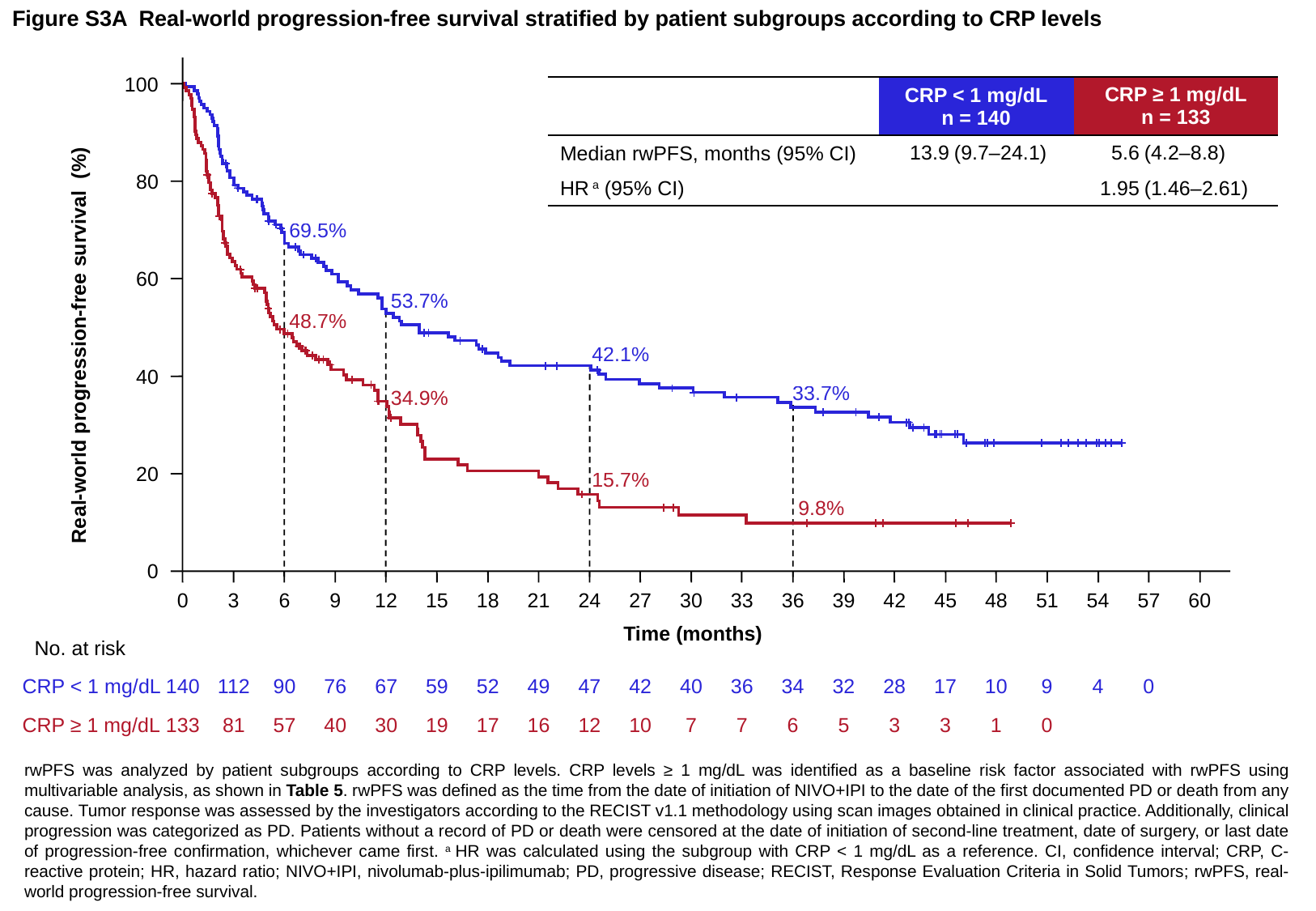

Figure S3A Real-world progression-free survival stratified by patient subgroups according to CRP levels
100
| | CRP < 1 mg/dL n = 140 | | CRP ≥ 1 mg/dL n = 133 | |
| --- | --- | --- | --- | --- |
| Median rwPFS, months (95% CI) | 13.9 | (9.7–24.1) | 5.6 | (4.2–8.8) |
| HR a (95% CI) | | | 1.95 | (1.46–2.61) |
80
69.5%
60
53.7%
48.7%
Real-world progression-free survival (%)
42.1%
40
33.7%
34.9%
20
15.7%
9.8%
0
0
3
6
9
12
15
18
21
24
27
30
33
36
39
42
45
48
51
54
57
60
Time (months)
No. at risk
CRP < 1 mg/dL
140
112
90
76
67
59
52
49
47
42
40
36
34
32
28
17
10
9
4
0
CRP ≥ 1 mg/dL
133
81
57
40
30
19
17
16
12
10
7
7
6
5
3
3
1
0
rwPFS was analyzed by patient subgroups according to CRP levels. CRP levels ≥ 1 mg/dL was identified as a baseline risk factor associated with rwPFS using multivariable analysis, as shown in Table 5. rwPFS was defined as the time from the date of initiation of NIVO+IPI to the date of the first documented PD or death from any cause. Tumor response was assessed by the investigators according to the RECIST v1.1 methodology using scan images obtained in clinical practice. Additionally, clinical progression was categorized as PD. Patients without a record of PD or death were censored at the date of initiation of second-line treatment, date of surgery, or last date of progression-free confirmation, whichever came first. a HR was calculated using the subgroup with CRP < 1 mg/dL as a reference. CI, confidence interval; CRP, C-reactive protein; HR, hazard ratio; NIVO+IPI, nivolumab-plus-ipilimumab; PD, progressive disease; RECIST, Response Evaluation Criteria in Solid Tumors; rwPFS, real-world progression-free survival.

## Slide 5
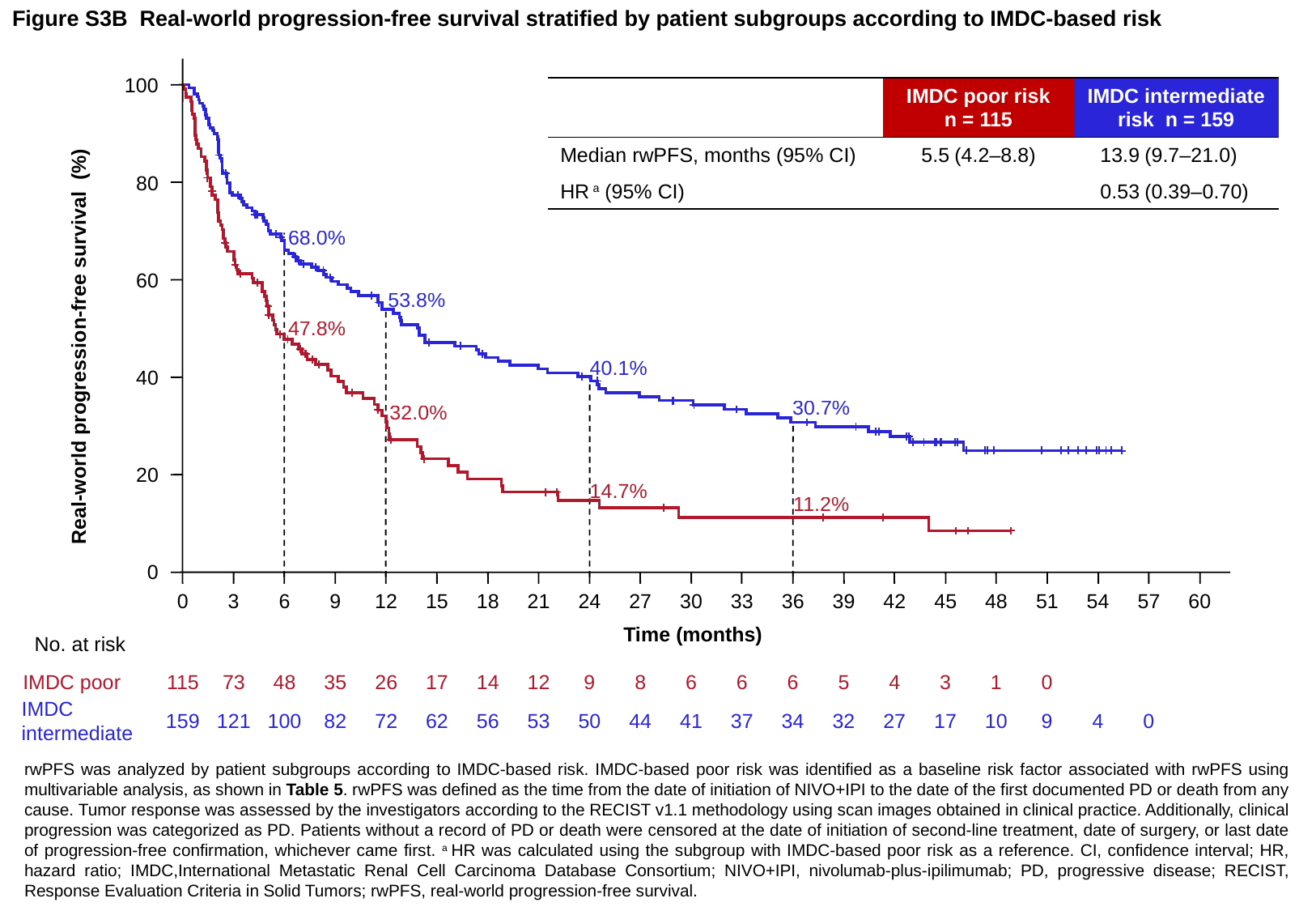

Figure S3B Real-world progression-free survival stratified by patient subgroups according to IMDC-based risk
100
| | IMDC poor risk n = 115 | | IMDC intermediate risk n = 159 | |
| --- | --- | --- | --- | --- |
| Median rwPFS, months (95% CI) | 5.5 | (4.2–8.8) | 13.9 | (9.7–21.0) |
| HR a (95% CI) | | | 0.53 | (0.39–0.70) |
80
68.0%
60
53.8%
47.8%
Real-world progression-free survival (%)
40.1%
40
30.7%
32.0%
20
14.7%
11.2%
0
0
3
6
9
12
15
18
21
24
27
30
33
36
39
42
45
48
51
54
57
60
Time (months)
No. at risk
IMDC poor
115
73
48
35
26
17
14
12
9
8
6
6
6
5
4
3
1
0
IMDC
intermediate
159
121
100
82
72
62
56
53
50
44
41
37
34
32
27
17
10
9
4
0
rwPFS was analyzed by patient subgroups according to IMDC-based risk. IMDC-based poor risk was identified as a baseline risk factor associated with rwPFS using multivariable analysis, as shown in Table 5. rwPFS was defined as the time from the date of initiation of NIVO+IPI to the date of the first documented PD or death from any cause. Tumor response was assessed by the investigators according to the RECIST v1.1 methodology using scan images obtained in clinical practice. Additionally, clinical progression was categorized as PD. Patients without a record of PD or death were censored at the date of initiation of second-line treatment, date of surgery, or last date of progression-free confirmation, whichever came first. a HR was calculated using the subgroup with IMDC-based poor risk as a reference. CI, confidence interval; HR, hazard ratio; IMDC,International Metastatic Renal Cell Carcinoma Database Consortium; NIVO+IPI, nivolumab-plus-ipilimumab; PD, progressive disease; RECIST, Response Evaluation Criteria in Solid Tumors; rwPFS, real-world progression-free survival.

## Slide 6
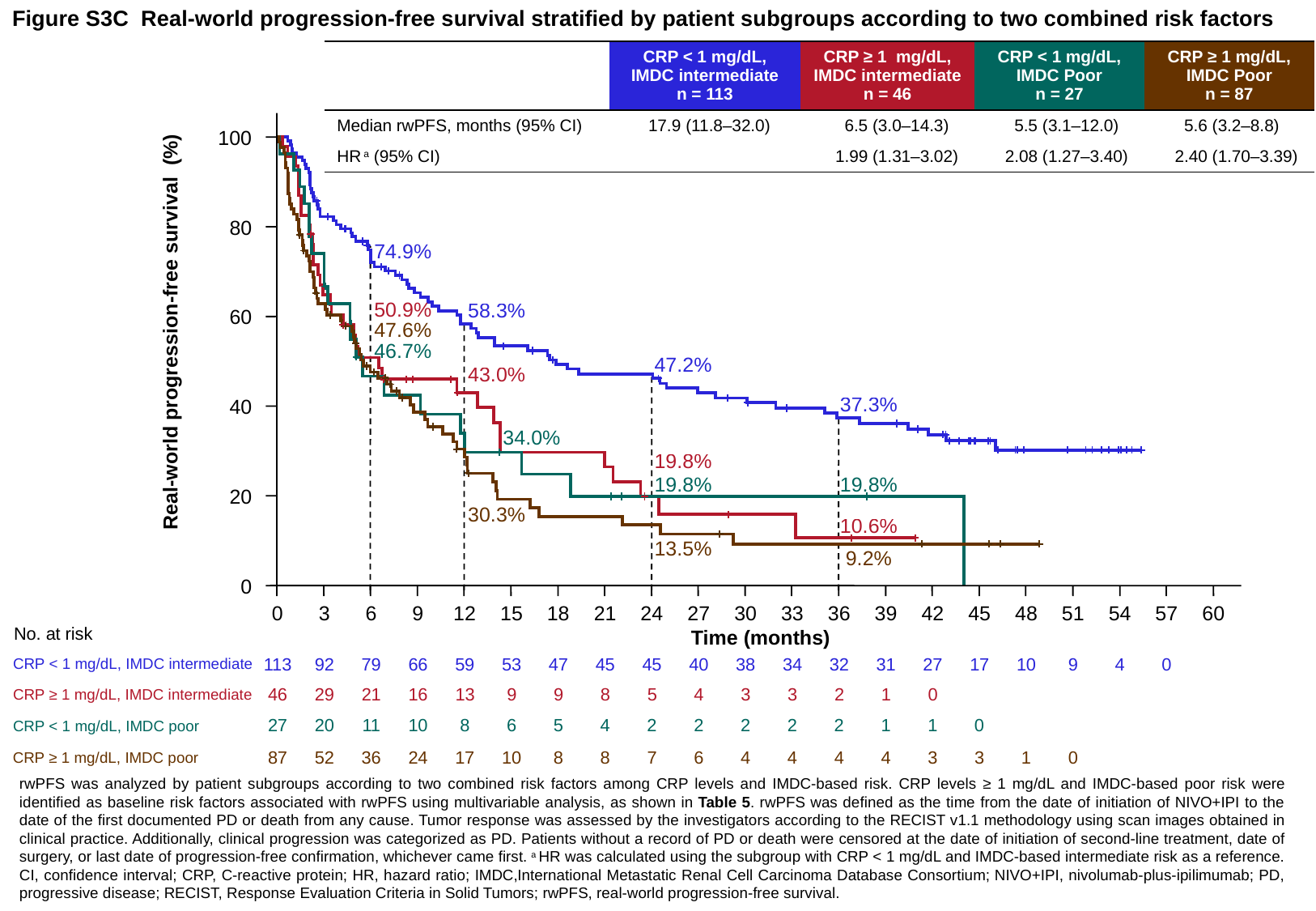

Figure S3C Real-world progression-free survival stratified by patient subgroups according to two combined risk factors
| | CRP < 1 mg/dL, IMDC intermediate n = 113 | | CRP ≥ 1 mg/dL, IMDC intermediate n = 46 | | CRP < 1 mg/dL, IMDC Poor n = 27 | | CRP ≥ 1 mg/dL, IMDC Poor n = 87 | |
| --- | --- | --- | --- | --- | --- | --- | --- | --- |
| Median rwPFS, months (95% CI) | 17.9 | (11.8–32.0) | 6.5 | (3.0–14.3) | 5.5 | (3.1–12.0) | 5.6 | (3.2–8.8) |
| HR a (95% CI) | | | 1.99 | (1.31–3.02) | 2.08 | (1.27–3.40) | 2.40 | (1.70–3.39) |
100
80
74.9%
50.9%
58.3%
60
47.6%
Real-world progression-free survival (%)
46.7%
47.2%
43.0%
37.3%
40
34.0%
19.8%
19.8%
19.8%
20
30.3%
10.6%
13.5%
9.2%
0
0
3
6
9
12
15
18
21
24
27
30
33
36
39
42
45
48
51
54
57
60
No. at risk
Time (months)
113
92
79
66
59
53
47
45
45
40
38
34
32
31
27
17
10
9
4
0
CRP < 1 mg/dL, IMDC intermediate
CRP ≥ 1 mg/dL, IMDC intermediate
46
29
21
16
13
9
9
8
5
4
3
3
2
1
0
27
20
11
10
8
6
5
4
2
2
2
2
2
1
1
0
CRP < 1 mg/dL, IMDC poor
CRP ≥ 1 mg/dL, IMDC poor
87
52
36
24
17
10
8
8
7
6
4
4
4
4
3
3
1
0
rwPFS was analyzed by patient subgroups according to two combined risk factors among CRP levels and IMDC-based risk. CRP levels ≥ 1 mg/dL and IMDC-based poor risk were identified as baseline risk factors associated with rwPFS using multivariable analysis, as shown in Table 5. rwPFS was defined as the time from the date of initiation of NIVO+IPI to the date of the first documented PD or death from any cause. Tumor response was assessed by the investigators according to the RECIST v1.1 methodology using scan images obtained in clinical practice. Additionally, clinical progression was categorized as PD. Patients without a record of PD or death were censored at the date of initiation of second-line treatment, date of surgery, or last date of progression-free confirmation, whichever came first. a HR was calculated using the subgroup with CRP < 1 mg/dL and IMDC-based intermediate risk as a reference. CI, confidence interval; CRP, C-reactive protein; HR, hazard ratio; IMDC,International Metastatic Renal Cell Carcinoma Database Consortium; NIVO+IPI, nivolumab-plus-ipilimumab; PD, progressive disease; RECIST, Response Evaluation Criteria in Solid Tumors; rwPFS, real-world progression-free survival.

## Slide 7
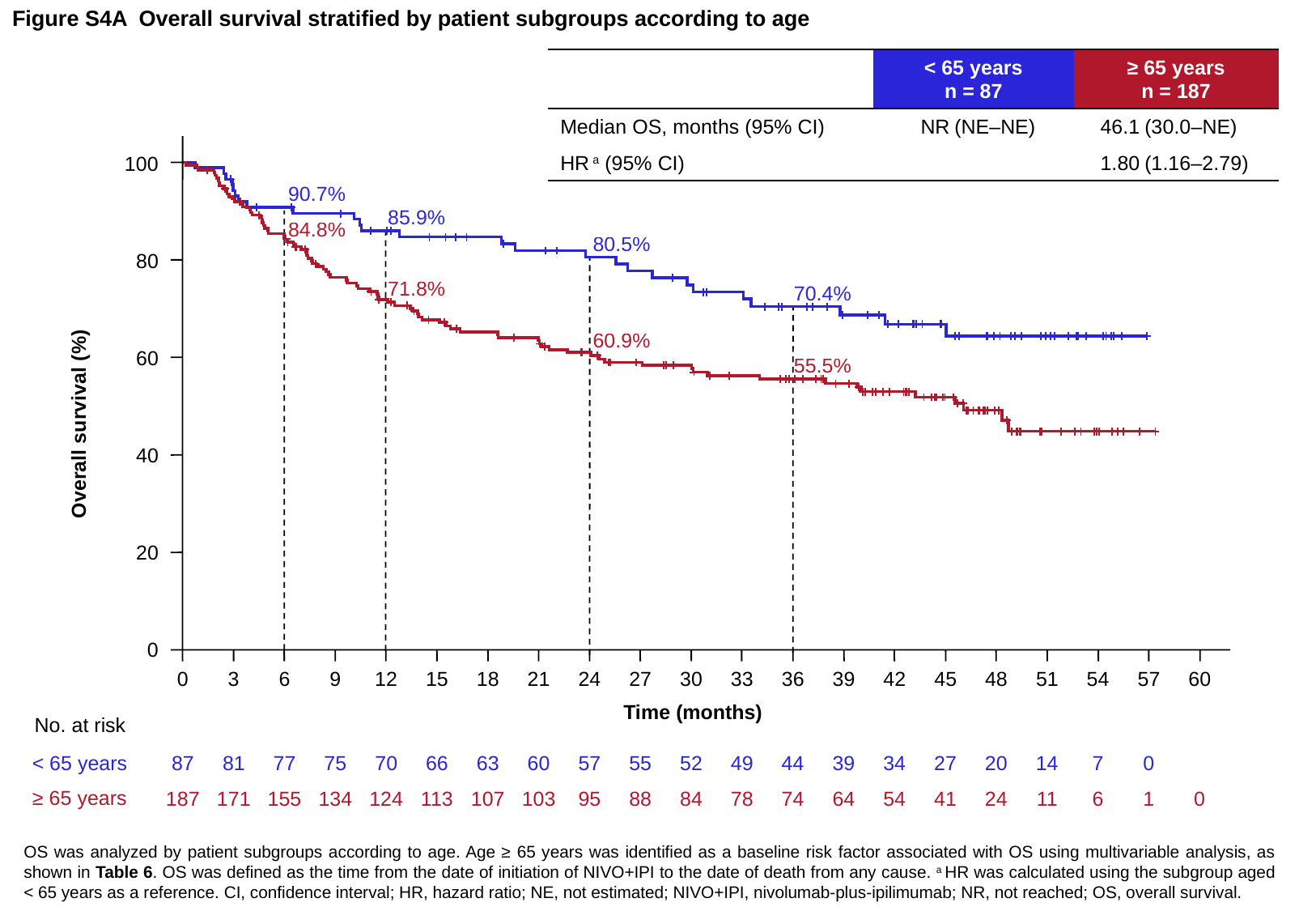

Figure S4A Overall survival stratified by patient subgroups according to age
| | < 65 years n = 87 | | ≥ 65 years n = 187 | |
| --- | --- | --- | --- | --- |
| Median OS, months (95% CI) | NR | (NE–NE) | 46.1 | (30.0–NE) |
| HR a (95% CI) | | | 1.80 | (1.16–2.79) |
100
90.7%
85.9%
84.8%
80.5%
80
71.8%
70.4%
60.9%
60
55.5%
Overall survival (%)
40
20
0
0
3
6
9
12
15
18
21
24
27
30
33
36
39
42
45
48
51
54
57
60
Time (months)
No. at risk
< 65 years
87
81
77
75
70
66
63
60
57
55
52
49
44
39
34
27
20
14
7
0
≥ 65 years
187
171
155
134
124
113
107
103
95
88
84
78
74
64
54
41
24
11
6
1
0
OS was analyzed by patient subgroups according to age. Age ≥ 65 years was identified as a baseline risk factor associated with OS using multivariable analysis, as shown in Table 6. OS was defined as the time from the date of initiation of NIVO+IPI to the date of death from any cause. a HR was calculated using the subgroup aged < 65 years as a reference. CI, confidence interval; HR, hazard ratio; NE, not estimated; NIVO+IPI, nivolumab-plus-ipilimumab; NR, not reached; OS, overall survival.

## Slide 8
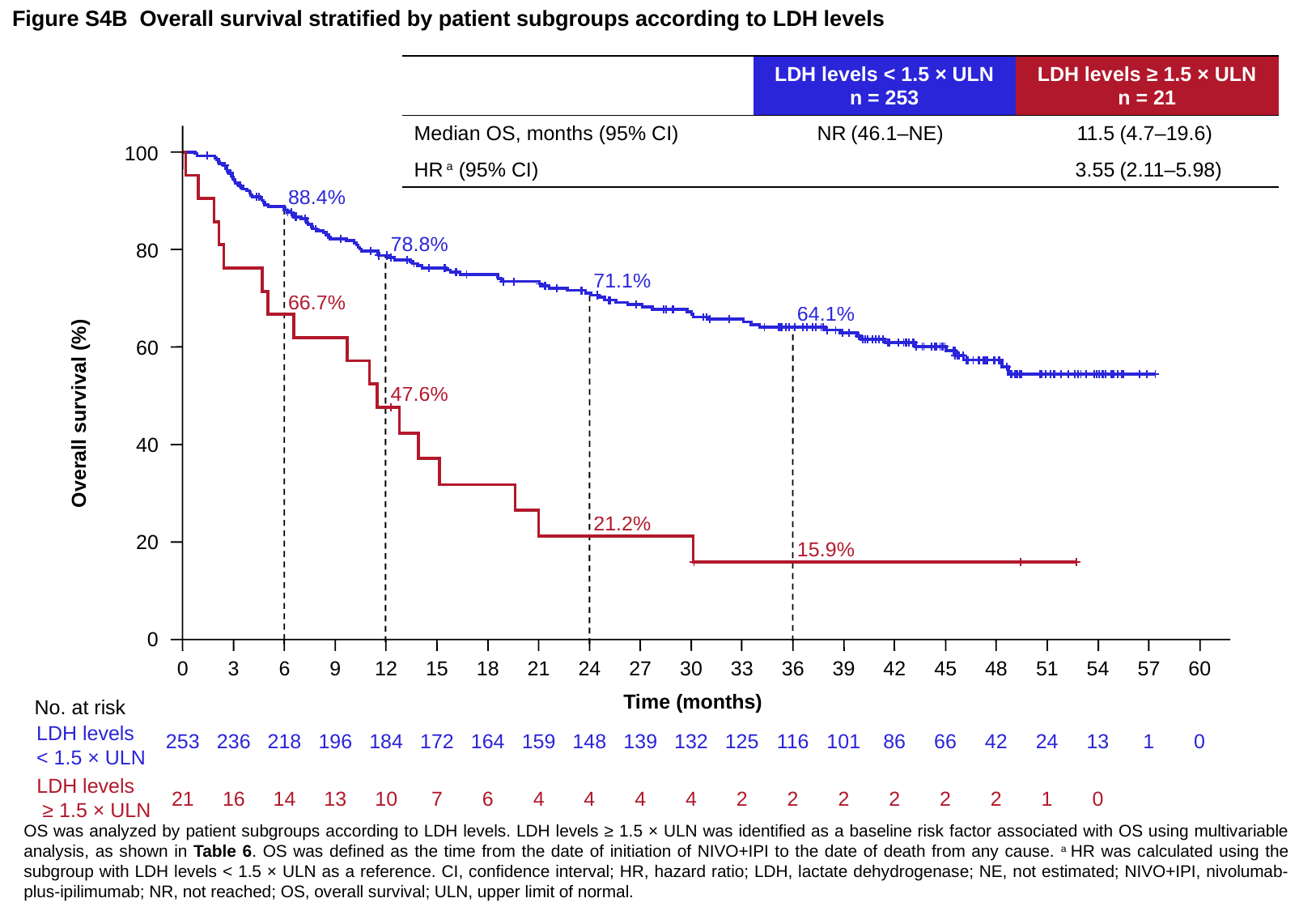

Figure S4B Overall survival stratified by patient subgroups according to LDH levels
| | LDH levels < 1.5 × ULN n = 253 | | LDH levels ≥ 1.5 × ULN n = 21 | |
| --- | --- | --- | --- | --- |
| Median OS, months (95% CI) | NR | (46.1–NE) | 11.5 | (4.7–19.6) |
| HR a (95% CI) | | | 3.55 | (2.11–5.98) |
100
88.4%
78.8%
80
71.1%
66.7%
64.1%
60
47.6%
Overall survival (%)
40
21.2%
20
15.9%
0
0
3
6
9
12
15
18
21
24
27
30
33
36
39
42
45
48
51
54
57
60
Time (months)
No. at risk
LDH levels
< 1.5 × ULN
0
253
236
218
196
184
172
164
159
148
139
132
125
116
101
86
66
42
24
13
1
LDH levels
 ≥ 1.5 × ULN
21
16
14
13
10
7
6
4
4
4
4
2
2
2
2
2
2
1
0
OS was analyzed by patient subgroups according to LDH levels. LDH levels ≥ 1.5 × ULN was identified as a baseline risk factor associated with OS using multivariable analysis, as shown in Table 6. OS was defined as the time from the date of initiation of NIVO+IPI to the date of death from any cause. a HR was calculated using the subgroup with LDH levels < 1.5 × ULN as a reference. CI, confidence interval; HR, hazard ratio; LDH, lactate dehydrogenase; NE, not estimated; NIVO+IPI, nivolumab-plus-ipilimumab; NR, not reached; OS, overall survival; ULN, upper limit of normal.

## Slide 9
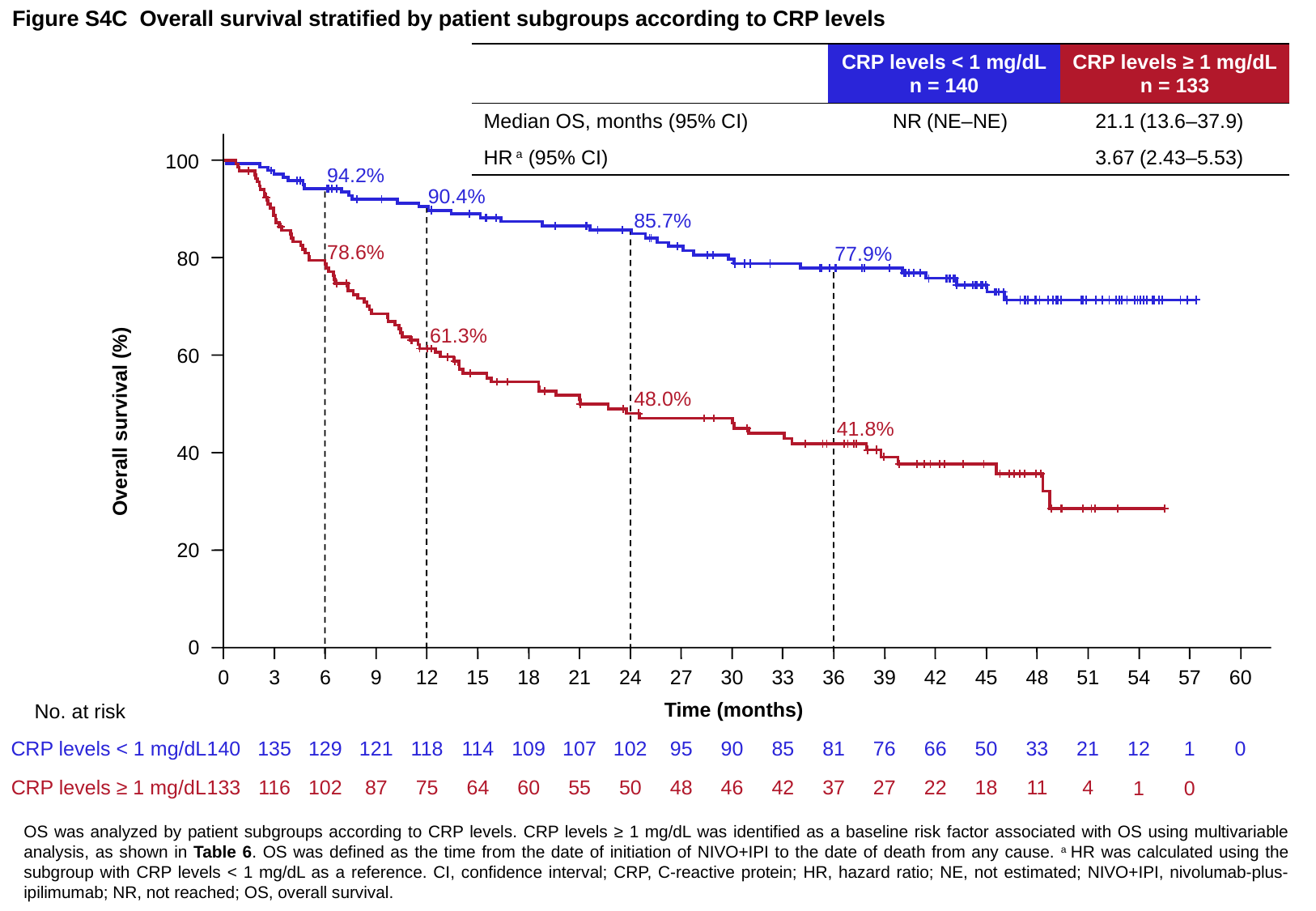

Figure S4C Overall survival stratified by patient subgroups according to CRP levels
| | CRP levels < 1 mg/dL n = 140 | | CRP levels ≥ 1 mg/dL n = 133 | |
| --- | --- | --- | --- | --- |
| Median OS, months (95% CI) | NR | (NE–NE) | 21.1 | (13.6–37.9) |
| HR a (95% CI) | | | 3.67 | (2.43–5.53) |
100
94.2%
90.4%
85.7%
78.6%
77.9%
80
61.3%
60
48.0%
Overall survival (%)
41.8%
40
20
0
0
3
6
9
12
15
18
21
24
27
30
33
36
39
42
45
48
51
54
57
60
Time (months)
No. at risk
CRP levels < 1 mg/dL
0
140
135
129
121
118
114
109
107
102
95
90
85
81
76
66
50
33
21
12
1
CRP levels ≥ 1 mg/dL
133
116
102
87
75
64
60
55
50
48
46
42
37
27
22
18
11
4
1
0
OS was analyzed by patient subgroups according to CRP levels. CRP levels ≥ 1 mg/dL was identified as a baseline risk factor associated with OS using multivariable analysis, as shown in Table 6. OS was defined as the time from the date of initiation of NIVO+IPI to the date of death from any cause. a HR was calculated using the subgroup with CRP levels < 1 mg/dL as a reference. CI, confidence interval; CRP, C-reactive protein; HR, hazard ratio; NE, not estimated; NIVO+IPI, nivolumab-plus-ipilimumab; NR, not reached; OS, overall survival.

## Slide 10
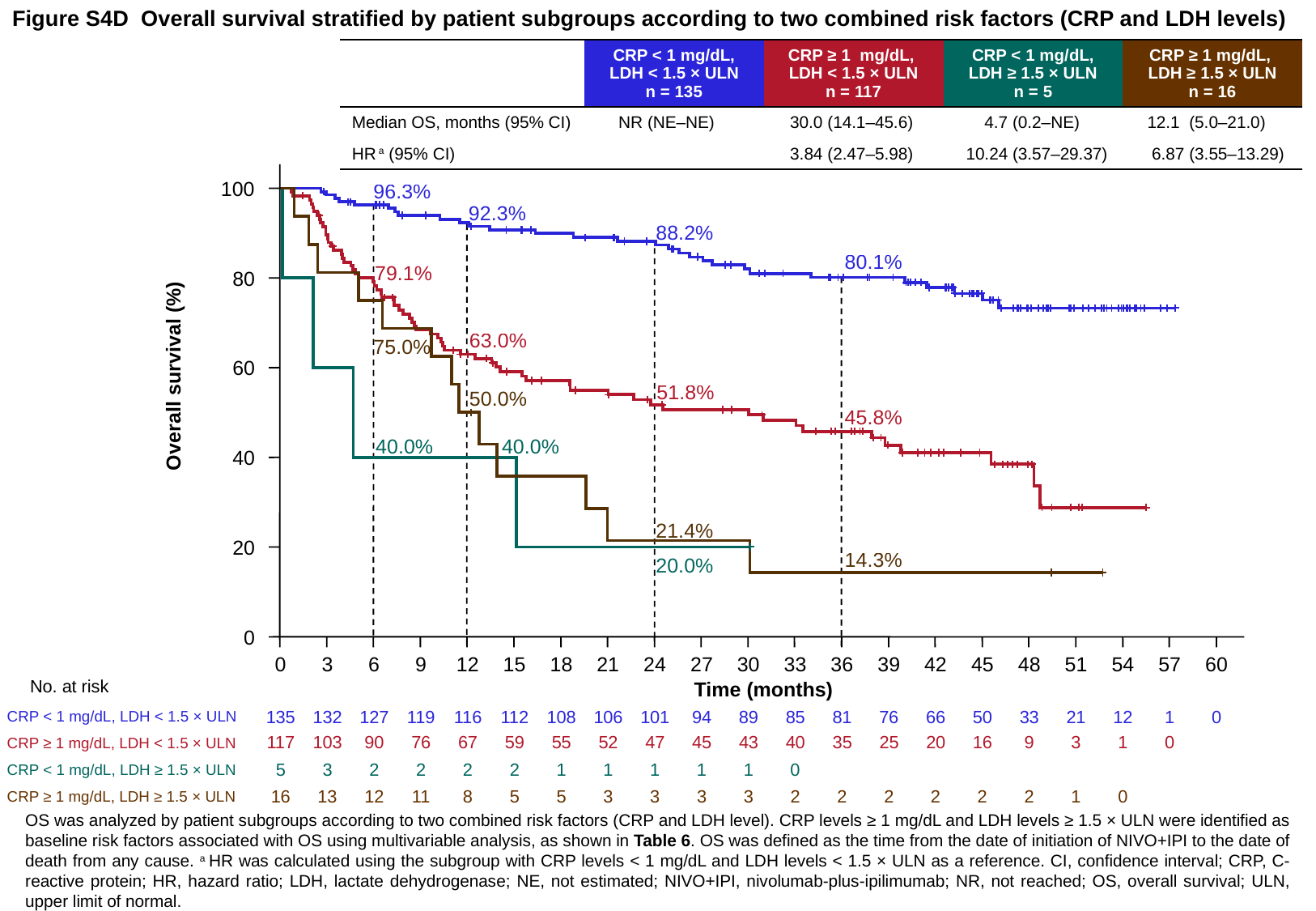

Figure S4D Overall survival stratified by patient subgroups according to two combined risk factors (CRP and LDH levels)
| | CRP < 1 mg/dL, LDH < 1.5 × ULN n = 135 | | CRP ≥ 1 mg/dL, LDH < 1.5 × ULN n = 117 | | CRP < 1 mg/dL, LDH ≥ 1.5 × ULN n = 5 | | CRP ≥ 1 mg/dL, LDH ≥ 1.5 × ULN n = 16 | |
| --- | --- | --- | --- | --- | --- | --- | --- | --- |
| Median OS, months (95% CI) | NR | (NE–NE) | 30.0 | (14.1–45.6) | 4.7 | (0.2–NE) | 12.1 | (5.0–21.0) |
| HR a (95% CI) | | | 3.84 | (2.47–5.98) | 10.24 | (3.57–29.37) | 6.87 | (3.55–13.29) |
100
96.3%
92.3%
88.2%
80.1%
79.1%
80
63.0%
75.0%
60
Overall survival (%)
51.8%
50.0%
45.8%
40.0%
40.0%
40
21.4%
20
14.3%
20.0%
0
0
3
6
9
12
15
18
21
24
27
30
33
36
39
42
45
48
51
54
57
60
No. at risk
Time (months)
135
132
127
119
116
112
108
106
101
94
89
85
81
76
66
50
33
21
12
1
CRP < 1 mg/dL, LDH < 1.5 × ULN
0
CRP ≥ 1 mg/dL, LDH < 1.5 × ULN
117
103
90
76
67
59
55
52
47
45
43
40
35
25
20
16
9
3
1
0
5
3
2
2
2
2
1
1
1
1
1
0
CRP < 1 mg/dL, LDH ≥ 1.5 × ULN
16
13
12
11
8
5
5
3
3
3
3
2
2
2
2
2
2
1
0
CRP ≥ 1 mg/dL, LDH ≥ 1.5 × ULN
OS was analyzed by patient subgroups according to two combined risk factors (CRP and LDH level). CRP levels ≥ 1 mg/dL and LDH levels ≥ 1.5 × ULN were identified as baseline risk factors associated with OS using multivariable analysis, as shown in Table 6. OS was defined as the time from the date of initiation of NIVO+IPI to the date of death from any cause. a HR was calculated using the subgroup with CRP levels < 1 mg/dL and LDH levels < 1.5 × ULN as a reference. CI, confidence interval; CRP, C-reactive protein; HR, hazard ratio; LDH, lactate dehydrogenase; NE, not estimated; NIVO+IPI, nivolumab-plus-ipilimumab; NR, not reached; OS, overall survival; ULN, upper limit of normal.

## Slide 11
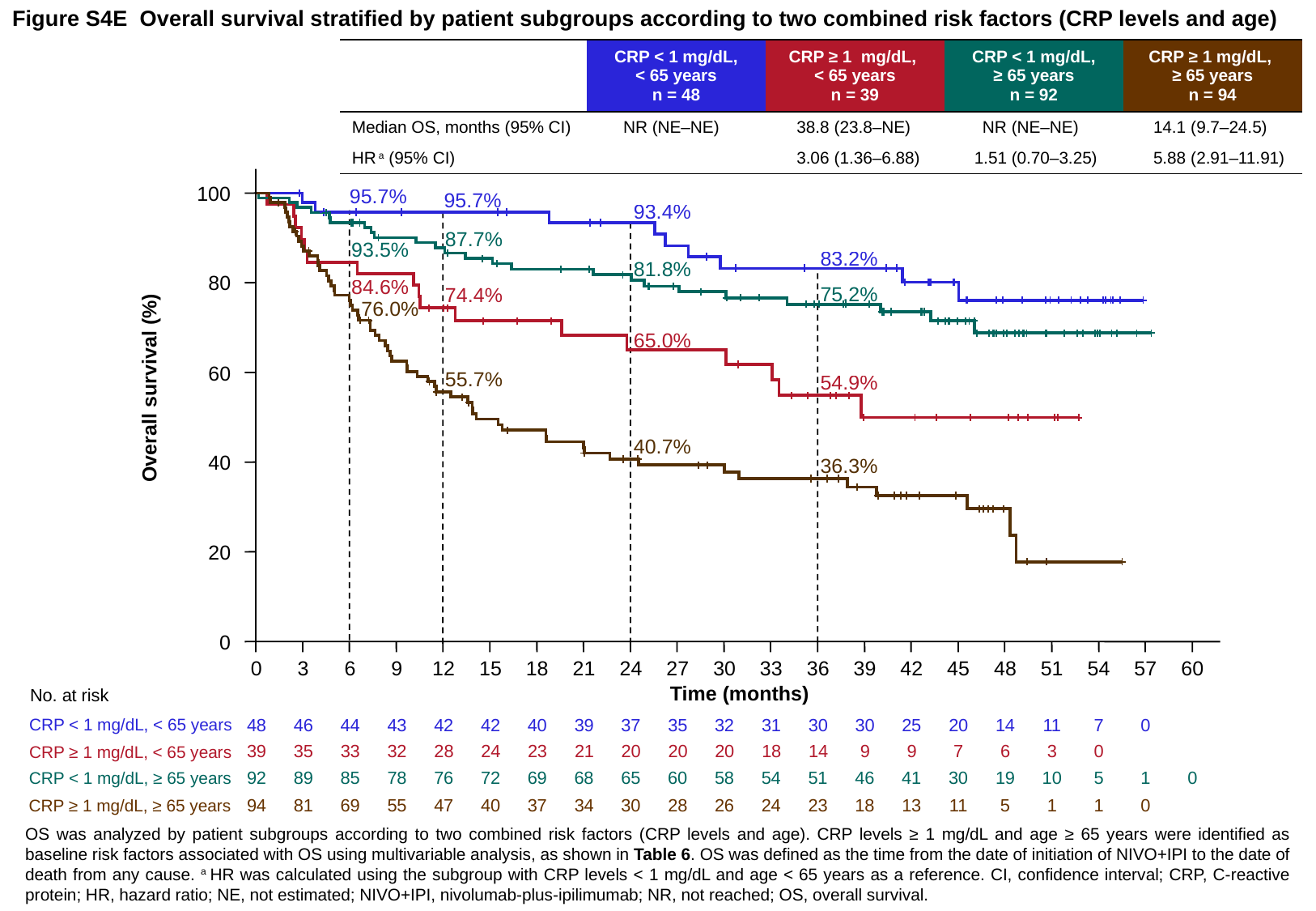

Figure S4E Overall survival stratified by patient subgroups according to two combined risk factors (CRP levels and age)
| | CRP < 1 mg/dL, < 65 years n = 48 | | CRP ≥ 1 mg/dL, < 65 years n = 39 | | CRP < 1 mg/dL, ≥ 65 years n = 92 | | CRP ≥ 1 mg/dL, ≥ 65 years n = 94 | |
| --- | --- | --- | --- | --- | --- | --- | --- | --- |
| Median OS, months (95% CI) | NR | (NE–NE) | 38.8 | (23.8–NE) | NR | (NE–NE) | 14.1 | (9.7–24.5) |
| HR a (95% CI) | | | 3.06 | (1.36–6.88) | 1.51 | (0.70–3.25) | 5.88 | (2.91–11.91) |
100
95.7%
95.7%
93.4%
87.7%
93.5%
83.2%
81.8%
80
84.6%
75.2%
74.4%
76.0%
65.0%
60
55.7%
54.9%
Overall survival (%)
40.7%
40
36.3%
20
0
0
3
6
9
12
15
18
21
24
27
30
33
36
39
42
45
48
51
54
57
60
Time (months)
No. at risk
48
46
44
43
42
42
40
39
37
35
32
31
30
30
25
20
14
11
7
0
CRP < 1 mg/dL, < 65 years
CRP ≥ 1 mg/dL, < 65 years
39
35
33
32
28
24
23
21
20
20
20
18
14
9
9
7
6
3
0
92
89
85
78
76
72
69
68
65
60
58
54
51
46
41
30
19
10
5
1
0
CRP < 1 mg/dL, ≥ 65 years
CRP ≥ 1 mg/dL, ≥ 65 years
94
81
69
55
47
40
37
34
30
28
26
24
23
18
13
11
5
1
1
0
OS was analyzed by patient subgroups according to two combined risk factors (CRP levels and age). CRP levels ≥ 1 mg/dL and age ≥ 65 years were identified as baseline risk factors associated with OS using multivariable analysis, as shown in Table 6. OS was defined as the time from the date of initiation of NIVO+IPI to the date of death from any cause. a HR was calculated using the subgroup with CRP levels < 1 mg/dL and age < 65 years as a reference. CI, confidence interval; CRP, C-reactive protein; HR, hazard ratio; NE, not estimated; NIVO+IPI, nivolumab-plus-ipilimumab; NR, not reached; OS, overall survival.

## Slide 12
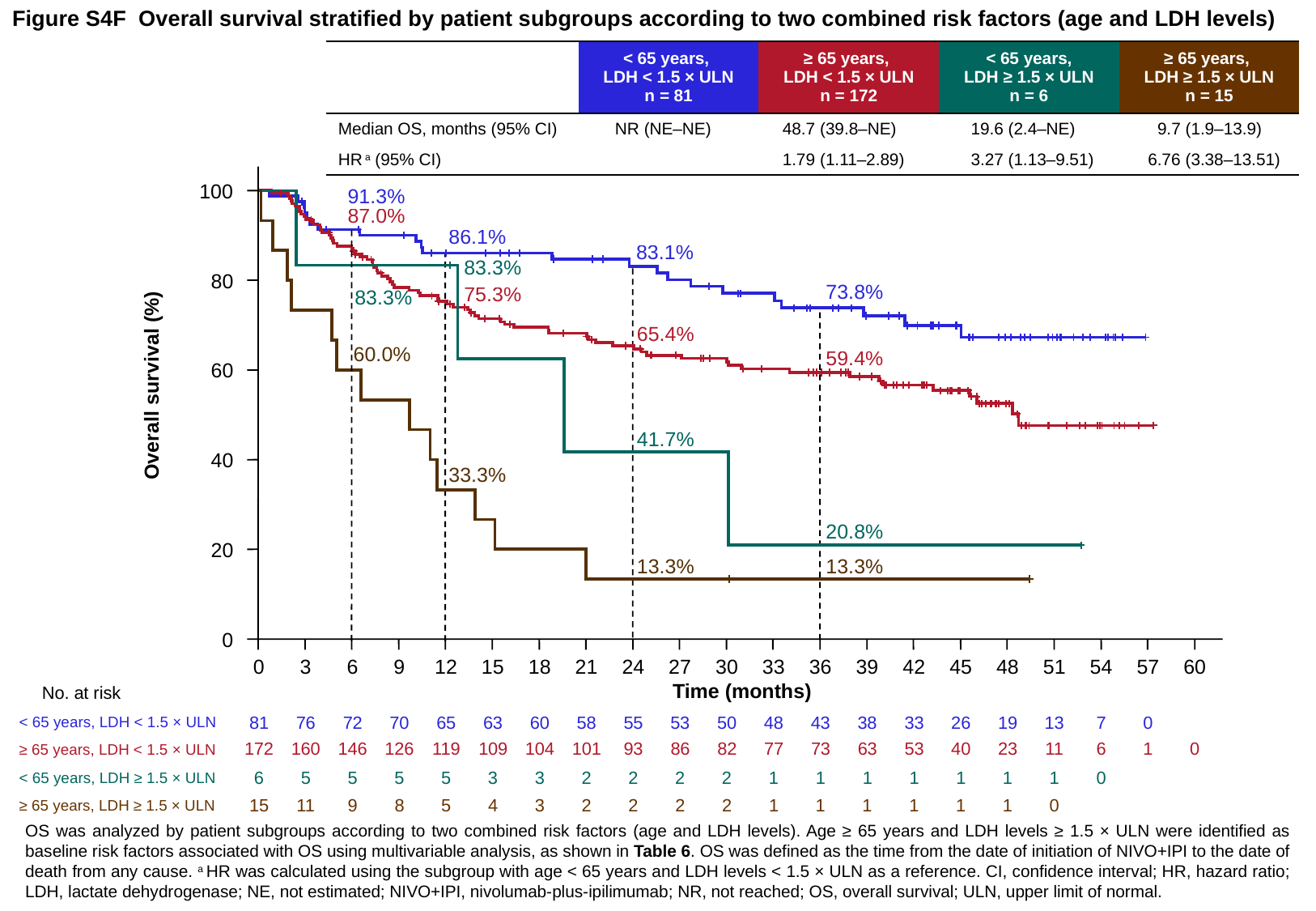

Figure S4F Overall survival stratified by patient subgroups according to two combined risk factors (age and LDH levels)
| | < 65 years, LDH < 1.5 × ULN n = 81 | | ≥ 65 years, LDH < 1.5 × ULN n = 172 | | < 65 years, LDH ≥ 1.5 × ULN n = 6 | | ≥ 65 years, LDH ≥ 1.5 × ULN n = 15 | |
| --- | --- | --- | --- | --- | --- | --- | --- | --- |
| Median OS, months (95% CI) | NR | (NE–NE) | 48.7 | (39.8–NE) | 19.6 | (2.4–NE) | 9.7 | (1.9–13.9) |
| HR a (95% CI) | | | 1.79 | (1.11–2.89) | 3.27 | (1.13–9.51) | 6.76 | (3.38–13.51) |
100
91.3%
87.0%
86.1%
83.1%
83.3%
80
73.8%
75.3%
83.3%
65.4%
60.0%
59.4%
60
Overall survival (%)
41.7%
40
33.3%
20.8%
20
13.3%
13.3%
0
0
3
6
9
12
15
18
21
24
27
30
33
36
39
42
45
48
51
54
57
60
Time (months)
No. at risk
81
76
72
70
65
63
60
58
55
53
50
48
43
38
33
26
19
13
7
0
< 65 years, LDH < 1.5 × ULN
≥ 65 years, LDH < 1.5 × ULN
172
160
146
126
119
109
104
101
93
86
82
77
73
63
53
40
23
11
6
1
0
6
5
5
5
5
3
3
2
2
2
2
1
1
1
1
1
1
1
0
< 65 years, LDH ≥ 1.5 × ULN
15
11
9
8
5
4
3
2
2
2
2
1
1
1
1
1
1
0
≥ 65 years, LDH ≥ 1.5 × ULN
OS was analyzed by patient subgroups according to two combined risk factors (age and LDH levels). Age ≥ 65 years and LDH levels ≥ 1.5 × ULN were identified as baseline risk factors associated with OS using multivariable analysis, as shown in Table 6. OS was defined as the time from the date of initiation of NIVO+IPI to the date of death from any cause. a HR was calculated using the subgroup with age < 65 years and LDH levels < 1.5 × ULN as a reference. CI, confidence interval; HR, hazard ratio; LDH, lactate dehydrogenase; NE, not estimated; NIVO+IPI, nivolumab-plus-ipilimumab; NR, not reached; OS, overall survival; ULN, upper limit of normal.
