# Supplementary material for: A Minimum 3‐Year Follow‐Up of Nivolumab‐Plus‐Ipilimumab in Japanese Patients With Advanced or Metastatic Renal Cell Carcinoma: A Final Analysis of the J‐ENCORE Study
Source: Int J Urol. 2026 Mar 17;33(3):e70400. doi: 10.1111/iju.70400 (PMC12993795; doi:10.1111/iju.70400)
Supplement: Supplementary file 2 — Table S1: List of investigators. Table S2: Summary of nivolumab‐plus‐ipilimumab discontinuation and subsequent therapy. [file IJU-33-0-s001.docx]

# **Supporting information**

Table S1 List of investigators

Table S2 Summary of nivolumab-plus-ipilimumab discontinuation and subsequent therapy

## **Table S1 List of investigators**

| Site name | Investigator |
| --- | --- |
| Sapporo Medical University Hospital | Naoya Masumori |
| Hokkaido University Hospital | Takashige Abe |
| Hirosaki University Hospital | Chikara Ohyama |
| Iwate Medical University Hospital | Wataru Obara |
| Yamagata University Hospital | Norihiko Tsuchiya |
| Akita University Hospital | Kazuyuki Numakura |
| University of Tsukuba Hospital | Hiroyuki Nishiyama |
| Saitama Medical University International Medical Center | Suguru Shirotake |
| Chiba Cancer Center | Atsushi Komaru |
| Chiba University Hospital | Tomohiko Ichikawa |
| Tokyo Women’s Medical University, Adachi Medical Center | Tsunenori Kondo |
| The Jikei University Hospital | Takahiro Kimura |
| Institute of Science Tokyo Hospital | Yasuhisa Fujii |
| Nippon Medical School Hospital | Yukihiro Kondo |
| Juntendo University Hospital | Shigeo Horie |
| The Cancer Institute Hospital of JFCR | Junji Yonese |
| Yokohama City University Hospital | Hisashi Hasumi |
| Kanagawa Cancer Center | Takeshi Kishida |
| Niigata University Medical & Dental Hospital | Yoshihiko Tomita |
| Toyama University Hospital | Hiroshi Kitamura |
| Kanazawa University Hospital | Atsushi Mizokami |
| Nagoya City University Hospital | Shuzo Hamamoto |
| Aichi Medical University Hospital | Naoto Sassa |
| Nara Medical University Hospital | Kiyohide Fujimoto |
| University Hospital, Kyoto Prefectural University of Medicine | Osamu Ukimura |
| Osaka International Cancer Institute | Kazuo Nishimura |
| Osaka University Hospital | Norio Nonomura |
| Kindai University Hospital | Hirotsugu Uemura |
| Kobe University Hospital | Tomoaki Terakawa |
| Hiroshima University Hospital | Nobuyuki Hinata |
| Okayama University Hospital | Satoshi Katayama |
| Yamaguchi University Hospital | Koji Shiraishi |
| Tokushima University Hospital | Masayuki Takahashi |
| Kagawa University Hospital | Mikio Sugimoto |
| Ehime University Hospital | Takashi Saika |
| Kyushu University Hospital | Masatoshi Eto |
| Nagasaki University Hospital | Kojiro Ohba |

## **Table S2 Summary of nivolumab-plus-ipilimumab discontinuation and subsequent therapy**

|  | Overall  n = 274 |
| --- | --- |
| Discontinuation of NIVO+IPI, n (%) | 249 (90.9) |
| Reasons for treatment discontinuation ^†^, n (%) |  |
| Discontinued due to PD | 118 (47.4) |
| Discontinued due to AE | 89 (35.7) |
| Discontinued due to other reasons ^‡^ | 66 (26.5) |
| With subsequent therapy, n (%) | 136 (49.6) |
| Subsequent therapy, n (%) |  |
| Cabozantinib | 93 (68.4) |
| Axitinib | 33 (24.3) |
| Others ^§^ | 10 (7.4) |

^†^ Multiple answers were allowed for reasons for treatment discontinuation. Only reasons for NIVO discontinuation were counted. ^‡^ Other reasons included good response, transfer to other hospital, personal reason, death, and others. ^§^ Others included pazopanib, sunitinib, nivolumab, and sorafenib. AE, adverse event; NIVO, nivolumab; NIVO+IPI, nivolumab-plus-ipilimumab; PD, progressive disease.
